# Neofunctionalization driven by positive selection led to the retention of the *loqs2* gene encoding an *Aedes* specific dsRNA binding protein

**DOI:** 10.1186/s12915-024-01821-4

**Published:** 2024-01-25

**Authors:** Carlos F. Estevez-Castro, Murillo F. Rodrigues, Antinéa Babarit, Flávia V. Ferreira, Elisa G. de Andrade, Eric Marois, Rodrigo Cogni, Eric R. G. R. Aguiar, João T. Marques, Roenick P. Olmo

**Affiliations:** 1 Department of Biochemistry and Immunology, Instituto de Ciências Biológicas, Universidade Federal de Minas Gerais, Belo Horizonte, 31270-901 Brazil; 2 CNRS UPR9022, Inserm U1257, Université de Strasbourg, 67084 Strasbourg, France; 3 Institute of Ecology and Evolution, University of Oregon, Eugene, OR 97403-5289 USA; 4 Department of Ecology, Institute of Biosciences, University of São Paulo, São Paulo, 05508-090 Brazil; 5 Department of Biological Science, Center of Biotechnology and Genetics, State University of Santa Cruz, Ilhéus, 45662-900 Brazil

**Keywords:** *loqs2*, RNA interference (RNAi), *Aedes* mosquitoes, Double-stranded RNA (dsRNA), dsRNA binding protein (dsRBP)

## Abstract

**Background:**

Mosquito borne viruses, such as dengue, Zika, yellow fever and Chikungunya, cause millions of infections every year. These viruses are mostly transmitted by two urban-adapted mosquito species, *Aedes aegypti* and *Aedes albopictus*. Although mechanistic understanding remains largely unknown, *Aedes* mosquitoes may have unique adaptations that lower the impact of viral infection. Recently, we reported the identification of an *Aedes* specific double-stranded RNA binding protein (dsRBP), named Loqs2, that is involved in the control of infection by dengue and Zika viruses in mosquitoes. Preliminary analyses suggested that the *loqs2* gene is a paralog of *loquacious* (*loqs*) and *r2d2*, two co-factors of the RNA interference (RNAi) pathway, a major antiviral mechanism in insects.

**Results:**

Here we analyzed the origin and evolution of *loqs2*. Our data suggest that *loqs2* originated from two independent duplications of the first double-stranded RNA binding domain of *loqs* that occurred before the origin of the *Aedes Stegomyia* subgenus, around 31 million years ago*.* We show that the *loqs2* gene is evolving under relaxed purifying selection at a faster pace than *loqs*, with evidence of neofunctionalization driven by positive selection. Accordingly, we observed that Loqs2 is localized mainly in the nucleus, different from R2D2 and both isoforms of Loqs that are cytoplasmic. In contrast to *r2d2* and *loqs*, *loqs2* expression is stage- and tissue-specific, restricted mostly to reproductive tissues in adult *Ae. aegypti* and *Ae. albopictus*. Transgenic mosquitoes engineered to express *loqs2* ubiquitously undergo developmental arrest at larval stages that correlates with massive dysregulation of gene expression without major effects on microRNAs or other endogenous small RNAs, classically associated with RNA interference.

**Conclusions:**

Our results uncover the peculiar origin and neofunctionalization of *loqs2* driven by positive selection. This study shows an example of unique adaptations in *Aedes* mosquitoes that could ultimately help explain their effectiveness as virus vectors.

**Supplementary Information:**

The online version contains supplementary material available at 10.1186/s12915-024-01821-4.

## Background

*Aedes aegypti* and *Aedes albopictus* are the major vectors for arthropod-borne viruses (arboviruses), such as yellow fever, dengue (DENV), Zika (ZIKV), and chikungunya (CHIKV) viruses. DENV alone is estimated to be responsible for approximately 400 million infections and 20,000 deaths per year worldwide [1]. Globalization, urbanization and climate change are contributing to the spread of *Aedes* mosquitoes to previously uncolonized regions impacting virus transmission and emergence of new arboviruses with potential to affect human health [2]. Despite the urgent need, there are no treatments or vaccines for most arboviral diseases [3]. Aggravating this scenario, *Ae. aegypti* and *Ae. albopictus* are not only well adapted to urban settings and anthropophilic feeding but also seem to be naturally more susceptible to arboviruses under laboratory conditions [4–7]. This intrinsic susceptibility to arboviruses could explain their exquisite vector competence [7]. One could reason that the absence of common markers is suggestive of divergence in the immune response between *Aedes* and closely related species [8–10].

RNA interference (RNAi) and, in particular, the small interfering RNA (siRNA) pathway, is a broad antiviral defense mechanism in insects [9, 11, 12]. Virus-derived double-stranded RNAs (dsRNA), common replication intermediates during the virus replication cycle, are processed by Dicer-2 (Dcr-2) into siRNAs that are subsequently loaded into the nuclease Argonaute-2 (AGO2) to form the RNA-induced silencing complex (RISC). This complex target and cleave complementary viral RNAs, thus inhibiting virus replication. RNAi co-factors such as the dsRNA binding proteins (dsRBPs) Loquacious (Loqs) and R2D2 are essential for the biogenesis and loading of small RNAs to the RISC complex [13]. Also, in the *Ae. aegypti* cell line Aag2, Loqs and R2D2 seem to act non-redundantly in the antiviral branch of the siRNA pathway suggesting that these proteins have specific roles during virus infection [14].

The arms race between viruses and antiviral siRNA pathway resulted in rapid evolutionary rates of genes such as *Dcr-2, AGO2* and *r2d2* in *Drosophila* and mosquito species, influencing the fixation of duplications and losses of RNAi related genes in multiple insects [15–17]. This evolutionary pattern resulted in great divergence and specialization of RNAi genes even between closely phylogenetically related species [18]. Interestingly, we recently identified *loqs2*, a novel dsRBP gene in *Aedes* mosquitoes that might be a product of this evolutionary driving force [19]*.* Silencing of *loqs2* lead to increased levels of DENV and ZIKV viruses during systemic infection in *Ae. aegypti* and, in addition, ectopic expression of *loqs2* in the midgut resulted in the control of DENV and ZIKV infection in the mosquito [19]. These results suggest that *loqs2* might have evolved to be a regulator of antiviral defense but the connection of Loqs2 with antiviral RNAi in *Aedes* mosquitoes remains unclear.

Here, we investigated the origin and functional evolution of *loqs2* and used genetic tools to provide insight on its biological role. Our results indicate that *loqs2* originated from two independent duplications of the first dsRNA binding domain (dsRBD) of *loqs* that occurred before the *Aedes Stegomyia* subgenus radiation. Evolutionary analyses showed that *loqs2* diversified under relaxed purifying selection with sites evolving under positive selection, indicative of neofunctionalization, and remains evolving at a faster pace than other RNAi components such as *loqs* and *Dcr-2* in *Aedes* mosquitoes. Interestingly, Loqs2 is localized in the nucleus while R2D2 and both Loqs isoforms -PA and -PB are cytoplasmic, supporting the hypothesis of neofunctionalization of Loqs2. We also observed that *loqs2* expression is downregulated during the initial hours of embryogenesis and display a biased germline expression pattern in *Ae. aegypti* and *Ae. albopictus* mosquitoes. Ectopic expression of *loqs2* in *Ae. aegypti* larval stages led to developmental arrest possibly due to broad downregulation of gene expression. However, only minor differences in the small RNA profile were observed when comparing to controls, suggesting that Loqs2 might function outside the scope of RNAi in our experimental conditions. Together, our results provide insight on the origin and neofunctionalization of *loqs2,* an *Aedes Stegomya* specific dsRBP with functions that goes beyond the RNAi pathway.

## Results

### *loqs2* is a paralog of *loqs* that originated in the ancestor of the *Aedes Stegomyia* subgenus

**Fig. 1:**
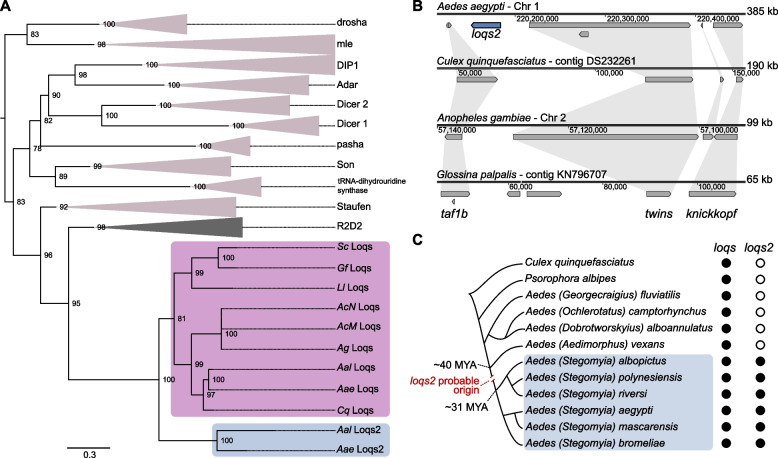
*loqs2* is a dsRBP paralog of *loqs* that originated in the ancestor of the *Aedes Stegomyia* subgenus. **A** Maximum likelihood phylogenetic tree constructed with the amino acid sequences of the dsRNA-binding proteins (dsRBPs) found in the genomes of *Ae. aegypti* (Aae)*, Ae. albopictus* (Aal), *C. quinquefasciatus (*Cq*), An. gambiae* (Ag), *An. coluzzii* Mali-NIH (AcM), *An. coluzzii* Ngousso (AcN), *L. longipalpis* Jacobina (Ll), *S. calcitrans* USDA (Sc) and *G. fuscipes* IAEA (Gf). Branches corresponding to the orthologs of drosha, mle, DIP1, Adar, Dicer-2, Dicer-1, pasha, Son, tRNA-dihydrouridine synthase, Staufen and R2D2 are collapsed to facilitate visualization. Loqs2 and Loqs clades are colored in grey and pink, respectively. Tree is rooted at the midpoint for visualization purposes. Node values correspond to the percentages of 1000 ultra-fast bootstrap iterations. Branch lengths represent substitutions per site. A non-collapsed tree is available in the Additional file 1: Fig. S1A. **B** Synteny analysis of *loqs2* flanking genomic regions among *Ae. aegypti, C. quinquefasciatus, An. gambiae* and *Glossina palpalis*. **C** Schematic cladogram indicating the probable origin of *loqs2*. The putative appearance of *loqs2* was inferred from the identification of sequences aligning to *loqs2* coding sequence in high-throughput sequencing data publicly available and are illustrated in the Additional file 1: Fig. S1B. Phylogenetic relationships and molecular clock were extracted from Soghigian et al. [21]

To infer the origin of *loqs2*, we screened the reference genomes of three hematophagous flies (*Lutzomyia longipalpis, Stomoxys calcitrans* and *Glossina fuscipes*) and five mosquitoes (*Ae. aegypti, Ae. albopictus, Culex quinquefasciatus, Anopheles gambiae* and *Anopheles coluzzii*) searching for protein coding sequences with at least one dsRBD (InterPRO accession IPR014720) [20] and determined their phylogenetic relationships at the protein level (Fig. 1A; extended version in the Additional file 1: Fig. S1A). The overall topology of the tree of dsRBPs shows monophyletic origins for each protein ortholog identified, with Loqs2 proteins located as a single sister clade of Loqs. These results confirm that *loqs2* is indeed a paralog of *loqs*, a co-factor of both microRNA and siRNA pathways in *Ae. aegypti* mosquitoes.

The evident diversity of the dsRBP protein family, as depicted by the predicted protein length, and number of dsRBDs in the species examined (Fig. 1A; extended version in the Additional file 1: Fig. S1A), suggests the presence of well-established and distinctive roles for these dsRBPs within the cell. Intriguingly, Loqs2 is the shortest among the identified dsRBPs from *Aedes* mosquitoes (Additional file 1: Fig. S1A), prompting the question of its evolutionary genomic origin. Considering that *loqs2* is exclusive to *Aedes* mosquitoes, we evaluated the corresponding genomic regions surrounding *loqs2* in other dipteran species (Fig. 1B). We observed synteny conservation in the region, in which three genes (*taf1b*, *twins*, and *knickkopf*) were present in all dipteran species analyzed. Other than the presence of *loqs2* in *Ae. aegypti,* we observed that two genes appeared between *taf1b* and *twins* in *G. palpalis*, both absent in the other dipterans studied. In addition, *An. gambiae*, *C. quinquefasciatus* and *Ae. aegypti* possesses an extra conserved gene inserted between *twins* and *knickkopf*, but absent in *G. palpalis*, suggesting that this region might be prone to gene insertions and deletions. Our analysis also highlights the large genomic expansion that *Ae. aegypti* has undergone in comparison to other dipteran species (Fig. 1B) [22]. The syntenic genomic region from the left and rightmost genes in *Ae. aegypti* is twice as large compared to *C. quinquefasciatus* and five times larger compared to *G. palpalis*.

To further trace the origin of *loqs2* in the *Aedes* genus, we analyzed publicly available high-throughput RNA-seq libraries and whole genome sequencing (WGS) data to inquire the presence of *loqs2* in four species belonging to different subgenera (*Georgecraigius, Ochlerotatus, Dobrotworskyius,* and *Aedimorphus*) and four species from the *Stegomyia* subgenus [23–27] (Additional file 2: Table S1). We also analyzed RNA-seq data from the mosquito *Psorophora albipes* [28], a species closely related to the *Aedes* genus (Fig. 1C) [21]. Using the *Ae. aegypti* and *Ae. albopictus* reference genomes for sequence alignment, we detected sequences that uniquely aligned to *loqs* gene from *Ae. aegypti* or *Ae. albopictus* in all nine species tested (Additional file 1: Fig. S1B). Due to the expected sequence diversity, particularly in the case of *loqs2,* we permitted multimapping with a high percentage of mismatches (50% of read length) and manually curated the alignments. Notably, we only retrieved sequences aligning to *Ae. aegypti* and *Ae. albopictus loqs2* genes from species belonging to the *Stegomyia* subgenus (Additional file 1: Fig. S1B). We also observed that sequences aligning to *loqs2* were scarcer and covered a smaller proportion of the gene in comparison to *loqs*. This observation could be attributed to lower gene expression of *loqs2* or a higher degree of sequence divergence of *loqs2* in the other species. Altogether, our results suggest that *loqs2* originated from a duplication of *loqs* that occurred before or at the radiation of the *Aedes Stegomyia* subgenus, estimated to have occurred around 31 million years ago (MYA) (Fig. 1C) [21].

### Two independent duplication events of the first dsRBD of *loqs* gave origin to *loqs2*

**Fig. 2:**
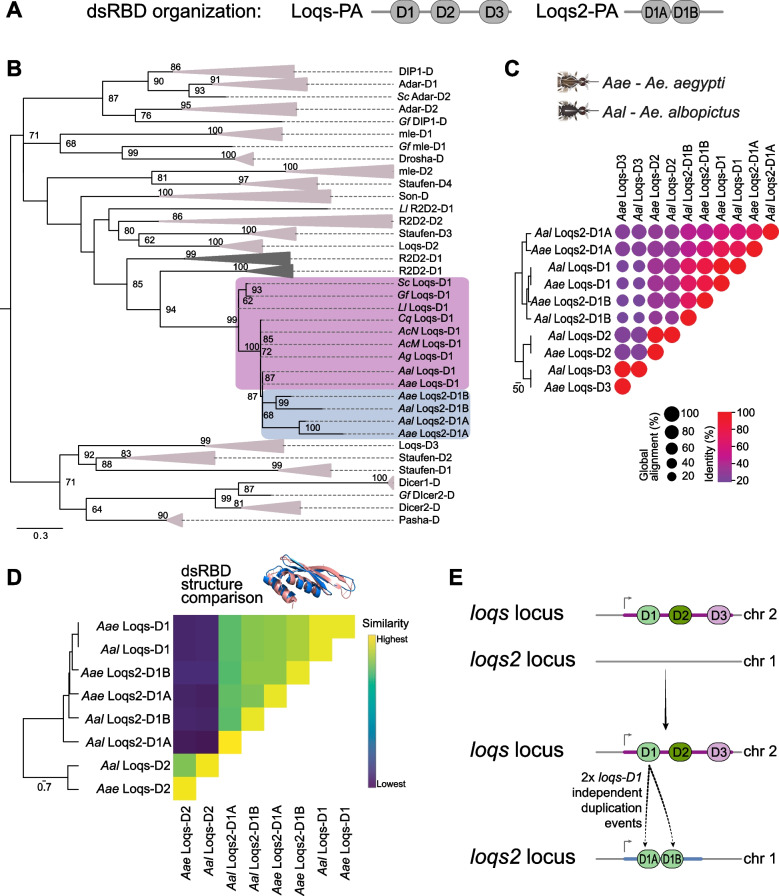
*loqs2* originated from two independent *loqs* dsRBD1 duplication events. **A** Organization of the double-stranded RNA binding domains (dsRBDs) of Loqs and Loqs2. **B** Phylogenetic relationships among the dsRBDs of the dsRBPs from Fig. 1A. Phylogeny was inferred by maximum likelihood and tree is rooted at the midpoint for visualization purposes. Branches corresponding to the dsRBDs from the orthology groups of Drosha, mle, DIP1, Adar, Dicer2, Dicer1, pasha, Son, Staufen R2D2 and Loqs-D2 and Loqs-D3 are collapsed to facilitate visualization. Loqs2 dsRBDs and Loqs-D1 clades are colored in grey and pink, respectively. Node values correspond to the percentages of 1000 ultra-fast bootstrap iterations. Node values > 60 are shown. Branch lengths represent substitutions per site. A non-collapsed tree is available on the Additional file 1: Fig. S2. **C** Correlation matrix showing EMBOSS Needle global identity and alignment percentages from the comparison between *Ae. aegypti* Loqs and Loqs2 dsRBD amino acid sequences. The dendrogram shows the level of similarity given by the distance matrix of identity percentages. **D** Heatmap showing the structural comparison between Loqs and Loqs2 dsRBD models generated using the DALI server. The dendrogram shows the average linkage clustering given by the similarity matrix. **E** putative model for *loqs2* origin

The *loqs2* coding sequence is expected to produce a small protein containing two canonical dsRBDs. However, the Loqs-PA differs from Loqs2-PA (Fig. 2A) by the distance between the first and second dsRBDs (D1 and D2, respectively) and the absence of a third dsRBD (D3), which is predicted to mediate protein–protein interaction and was shown to interact with Dicer-1 (Dcr-1) in *Drosophila* [29, 30]. Such differences prompted us to investigate the origins of *loqs2* by interrogating its structural features. First, we explored the phylogenetic relationships between the amino acid sequences of individual dsRBDs from Loqs and Loqs2 (Fig. 2B and Additional file 1: Fig. S2)*.* To better understand the relationship of Loqs2 domains with the evolutionary history of the dsRBPs*,* we included the dsRBDs of the dipteran dsRBPs from Fig. 1A (Prosite accession PS50137). Individual dsRBDs grouped in monophyletic clades for most dipterans, with exception of the DIP1 dsRBD from *G. fuscipes* and the R2D2-D1 from *L. longipalpis.* Overall, these results are in line with the assumption that dsRBPs evolved mainly by gene duplication and speciation [31]. In contrast to this model of origin, both dsRBDs of Loqs2 (D1A and D1B) clustered together with the first dsRBD of *Aedes* Loqs (D1), forming a monophyletic group that located inside the dipteran Loqs-D1 polytomy, and far from the dipteran Loqs-D2 monophyletic group*.* The observed polytomy is likely a result of the low phylogenetic distances within the dipteran Loqs-D1 group overshadowed by the broad phylogenetic divergence among the rest of dsRBDs. Nonetheless, the reliable node support of the nodes inside the polytomy suggests a branching pattern well supported by the data. We rescaled the branch lengths of the polytomy subtree to further examine its branching pattern (Additional file 1: Fig. S3A). The resulting cladogram positioned Loqs2’s dsRBDs (D1A and D1B) as the sister clade of the *Aedes* Loqs-D1 group, with reliable node support. However, it had limitations in clearly resolving the precise branching pattern between both dsRBDs of Loqs2, as indicated by the moderate support of the node. Global alignment of Loqs and Loqs2 dsRBDs from *Ae. aegypti* and *Ae. albopictus* (Fig. 2C) confirmed that both Loqs2-D1A and Loqs2-D1B were more similar to Loqs-D1 than to Loqs-D2. Importantly, these analyses also showed that both Loqs2-D1A and Loqs2-D1B are more similar to Loqs-D1 than to each other (Fig. 2C). We further compared structural properties of Loqs and Loqs2 dsRBDs using the resolved three-dimensional structures of the dsRBD1 and dsRBD2 of *D. melanogaster* Loqs-PD (PDB 5NPG and 5NPA, respectively) as reference for the modeling [32]. First, we predicted in silico the structures of individual dsRBDs of Loqs and Loqs2 from *Ae. aegypti* and *Ae. albopictus* and used the best model for structural comparisons using DALI [33]. Both dsRBDs of Loqs2 showed higher predicted structural similarity to Loqs-D1 (Fig. 2D). Notably, the second dsRBD of Loqs presented low structural similarity to all the other domains analyzed (*i.e.*, Loqs-D1, Loqs2-D1A and Loqs2-D1B). Taken together, our findings suggest that *loqs2* originated from two independent duplications of the first dsRBD of *loqs* and not from a direct gene duplication.

To further investigate this hypothesis at the genome level, we compared the exon–intron organization of both *loqs* and *loqs2* in the genomes of *Ae. aegypti* and *Ae. albopictus* (Additional file 1: Fig. S3B-C). The length and number of introns varied discreetly for both genes and species but the number of exon–intron boundaries spanning the first and second dsRBDs of *loqs* were similar (one intron inside D1 and no intron in D2). Interestingly, *loqs2* had one intron inside both dsRBD-1A and dsRBD-1B regions in *Ae. aegypti* or only one intron at dsRBD-1B of *Ae. albopictus*. The amino acid sequence present at the exon–intron-exon boundaries inside each dsRBD of *loqs2* is conserved and similar to the first dsRBD of *loqs* (Additional file 1: Fig. S3C), corroborating that *loqs2* originated from two duplications of *loqs* first dsRBD. We propose a model based on the *Ae. aegypti* genome organization, where *loqs*-D1 double duplications occurred from chromosome 2 towards chromosome 1, where *loqs2* is located (Fig. 2E).

### Positive selection shaped the evolution of *loqs2*

**Fig. 3:**
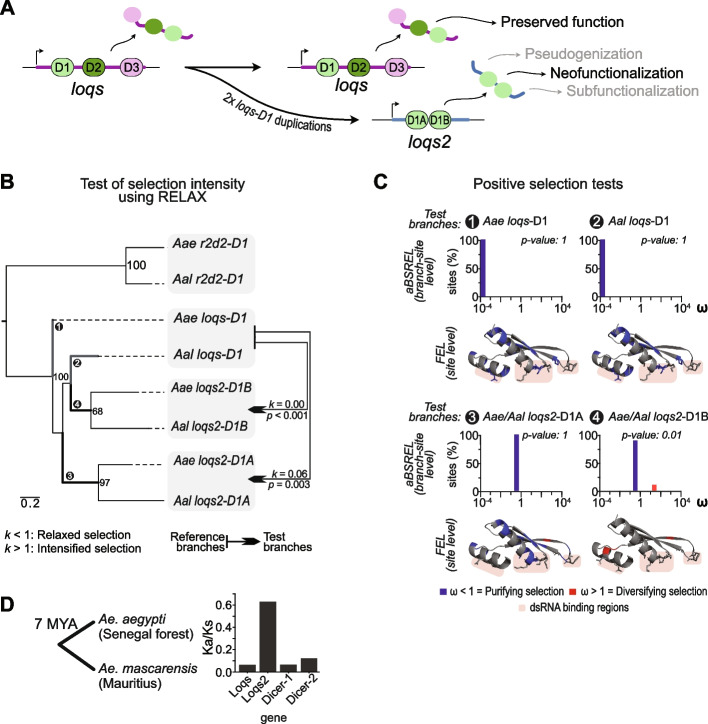
Positive selection shaped *loqs2* evolution. **A** Proposed evolutionary model followed by *loqs* and *loqs2* after the duplication events. In grey are written other common evolutionary models after any gene duplication. **B** Codon-based phylogenetic framework used to evaluate the selection forces acting on *loqs* and *loqs2* and test for relaxation or intensification of selection using RELAX. The maximum likelihood phylogenetic tree was constructed with the coding sequences of *loqs-D1, loqs2-D1A, loqs2-D1B* and *r2d2-D1* from *Ae. aegypti* (*Aae*) and *Ae. albopictus* (*Aal*). The tree is rooted at the midpoint for visualization purposes. Node values correspond to the percentages of 1000 ultra-fast bootstrap iterations. Node values > 60 are shown. Branch lengths represent nucleotide substitutions per codon site. RELAX selection intensity “*k”* values for each pairwise comparison between reference branches (arrow tails) and test branches (arrowheads) is represented in the tree. **C** Tests of positive selection based on the ω* (dN/dS)* metrics calculated from the phylogeny from **B**. The adaptive branch-site-model implemented in abSREL and the site-specific model implemented in FEL were used to test for selection among the numbered thick branches from the phylogeny in **B** (*loqs-D1* branches are colored in grey and *loqs2-D1* and *loqs2-D2* are in black). aBSREL results are represented by bar plots showing the ω value and percentage of sites for each ω class. Sites with significant evidence of purifying or diversifying selection determined by FEL are colored in the 3D models of Loqs-D1, Loqs2-D1 and Loqs2-D2 from *Ae. aegypti*. An alignment showing the sites with significant evidence of selection is available in the Additional file 1: Fig. S4. **D** Ka/Ks ratio calculated from the McDonald-Kreitman test (MKT) of *loqs*, *loqs2*, *Dicer-1* and *Dicer-2* from an *Ae. aegypti* population from a forest in Senegal compared to *Aedes mascarensis*. Divergence time between *Ae. aegypti* and *Ae. mascarensis* was taken from [23, 27]. The complete metrics reported from the MKT are available in the Additional file 2: Table S2**.** Statistical significance for RELAX (*p* > 0.05) in **B**, as well as aBSREL (*p* > 0.05) and FEL (*p* > 0.1) in **C** were determined using likelihood ratio tests. The *p*-value thresholds were maintained as recommended in the documentation of each test of selection

Gene duplications can have a range of outcomes. Typically, purifying selective constraints are expected to pressure the predecessor gene to maintain its original functions while the other is free to accumulate mutations. In this scenario, the most likely outcome is pseudogenization of the duplicated copy. Alternatively, accumulation of mutations can lead to subdivision of ancestral functions between duplicated genes, a process known as subfunctionalization. Occasionally, mutations may lead to the acquisition of a new function (neofunctionalization) that, if advantageous, will drive population fixation and diversification of the new gene through natural selection [34–36]. However, the evolution of *loqs2* is remarkable because it originated from two duplications of a single domain (*loqs-D1*) rather than the entire gene (Fig. 3A). One can speculate that the initial evolutionary history of *loqs2* occurred through neutral drift, possibly escaping pseudogenization by acquiring adaptive mutations that eventually rendered it functional. Because the key functions of *loqs* are conserved despite the presence of *loqs2* [14], we hypothesized that *loqs2* evolved to perform novel functions in the cell, *i.e.*, by neofunctionalization, with both dsRBDs playing a crucial role in the evolutionary trajectory of this gene.

To test for this hypothesis, we evaluated the selection forces that shaped the evolution of *loqs* and *loqs2* dsRBDs by using a set of methods based on the estimation of the ratio of nonsynonymous to synonymous substitution rates (ω or dN/dS) using a codon-based phylogenetic framework. This allowed us to build a topology by maximum likelihood inference, evaluating *loqs* and *loqs2* as ingroups and *r2d2* as an outgroup (Fig. 3B; Additional file 1: Fig. S4). First, we examined whether *loqs2* dsRBDs have evolved under a relaxed selection regime, i.e., close to neutrality (ω ≈ 1), in comparison to the selection regime of the first dsRBD of *loqs.* We measured this relaxation of selection using the RELAX software, which assesses shifts in the stringency of natural selection on a set of clades relative to a reference [37]. The RELAX test is based on the selection intensity parameter “*k”,* which establishes a comparison between the test and the reference branches of the phylogenetic tree (*k* > 1 indicates intensified selection while *k* < 1 indicates relaxed selection). We applied this strategy to each *Aedes loqs2* dsRBD clades as test branches compared to the first dsRBD of *loqs* as the reference branch. Our results showed significant evidence of relaxation of selective constraints (relaxed purifying selection) or weaker positive selection (relaxed positive selection) acting on each dsRBD of *loqs2* in comparison to *loqs*-D1 since *k* values were close to 0 in both cases (Fig. 3B).

Despite the reduced intensities of selection shaping the evolution of *loqs2* dsRBDs, we observed conservation of the amino acid motifs known to interact with dsRNA [38, 39] in both dsRBDs of *loqs2* in *Ae. aegypti* and *Ae. albopictus* (Additional file 1: Fig. S4). To identify signs of positive selection among the dsRBDs branches of *loqs* and *loqs2* we used aBSREL (adaptive Branch-Site Random Effects Likelihood). This selection test infers the optimal number of evolutionary rate categories (ω classes) among the branches [40] and models both site-level and branch-level evolutionary rate variation, further testing for positive selection. As aBSREL does not test for selection at specific sites, we complemented our branch-site-level approach with FEL (Fixed Effects Likelihood), a method that directly infers per-site selection (Fig. 3C). We applied both methodologies to the corresponding branches of the first dsRBD of *Ae. aegypti loqs* (*Aae loqs*-D1) or *Ae. albopictus loqs* (*Aal loqs*-D1), and both dsRBDs of *loqs2* in those species (highlighted in bold on Fig. 3B). The aBSREL test detected significant evidence of positive selection only in the second dsRBD of *loqs2* spanning 9% of sites (*loqs2*-D1B ω_2_ = 36.3) (Fig. 3C). In light of this analysis, we attribute the relaxation in selection detected by the RELAX software to a relaxation of purifying selection, as positive selection has been intensified as opposed by being relaxed. The ω classes of both dsRBDs were modeled close to neutrality (*loqs2*-D1A ω = 0.44 – 100% of sites; *loqs2*-D1B ω_1_ = 0.49 – 91% of sites, ω_2_ = 36.3 – 9% of sites) while the ω rate variations of *loqs-D1* for each *Aedes* species were modeled as nearly 0 for all sites. The FEL site-specific analysis identified a similar number of codons from the first dsRBD of *loqs* and *loqs2* with significant evidence of purifying selection (19 codons in *Aae loqs*-D1, 22 in *Aal loqs*-D1 and 18 in *loqs2*-D1A). However, no site under this evolutionary regime was detected in *loqs2*-D1B (Fig. 3C; Additional file 1: Fig. S4)*.* This test also revealed significant evidence of positive selection at one site of *loqs2*-D1 and three sites in *loqs2*-D1B. Notable, all of these sites (*loqs2*-D1A codon 24; *loqs2*-D1B codon 9, 17 and 24) are in close proximity to key residues involved in dsRNA interaction (Fig. 3C; Additional file 1: Fig. S4). Taken together, these findings suggest *loqs* is evolving under strong purifying selection (*loqs*-D1 ω < 0.001) while *loqs2* is evolving under less stringent purifying selection (*loqs2*-D1A ω = 0.44; *loqs2*-D1B ω_1_ = 0.49) with sites under positive selection (*loqs2*-D1B ω_2_ = 36.3). Furthermore, the location of the sites with significant evidence of positive selection suggests that Loqs2 dsRBDs might be still refining their dsRNA binding affinities.

Finally, we explored the recent evolutionary history of the *loqs2* gene including the dsRBDs and its flanking regions. We hypothesized that these flanking regions might have evolved faster than the dsRBDs given their non-genic origin. We tested for signs of positive selection using the McDonald and Kreitman test (MKT) available in the iMKT web server [41, 42]. The MKT infers the presence of recurrent positive selection by comparing polymorphic sites among a population to divergent sites between the population against an outgroup. We used publicly available exome data from *Ae. aegypti* samples from a forest in Senegal compared to *Ae. mascarensis* as an outgroup*,* a species that bifurcated from *Ae. aegypti* around 7 MYA [21, 23, 27]*.* To provide a comparative perspective, we also applied the same test to *Dcr-1* and *Dcr-2* genes, which are components of the microRNA and siRNA pathways, commonly used as hallmarks of purifying and diversifying selection among insects, including *Ae. aegypti* [16, 17]. The MKT did not detect significant evidence of positive selection for *loqs2*, or any of the genes tested (Additional file 2: Table S2) although this could be due to the low power of the test to detect weak selection [43, 44]*.* Nevertheless, *loqs2* has an increased number of nonsynonymous substitutions between species and also an increased number of nonsynonymous polymorphisms (Additional file 2: Table S2). This suggests that the high rate of evolution of *loqs2* is at least in part driven by a relaxation of purifying selection, providing support for our earlier conclusions based on codon substitution models. We also found that *loqs2* has an ω approximately 5 to 10 times higher than that calculated for *loqs* and other RNAi genes such as *Dcr-1* and *Dcr-2* (Fig. 3D)*.* Notably, in *D. melanogaster*, *Dcr-2*, *AGO2* and *r2d2* are rapidly evolving genes and display similar ω average values comparable to *loqs2* in *Ae. aegypti* [45]. Altogether, our results indicate that *loqs2* is diversifying faster than *loqs* in a recent evolutionary scale. These analyses reinforce the hypothesis that *loqs* and *loqs2* are evolving under different regimes of selection, with *loqs2* actively diversifying and fine tuning its new functions. Thus, *loqs2* could be performing roles (*e.g.*, antiviral immunity, control of gene expression) that *loqs* does not sustain, probably due in part to the strong evolutionary pressures that have constrained it within its shared role between the miRNA and the siRNA pathways [14]*.*

### *loqs2* cellular localization and expression pattern is distinct from *loqs* and *r2d2*

**Fig. 4:**
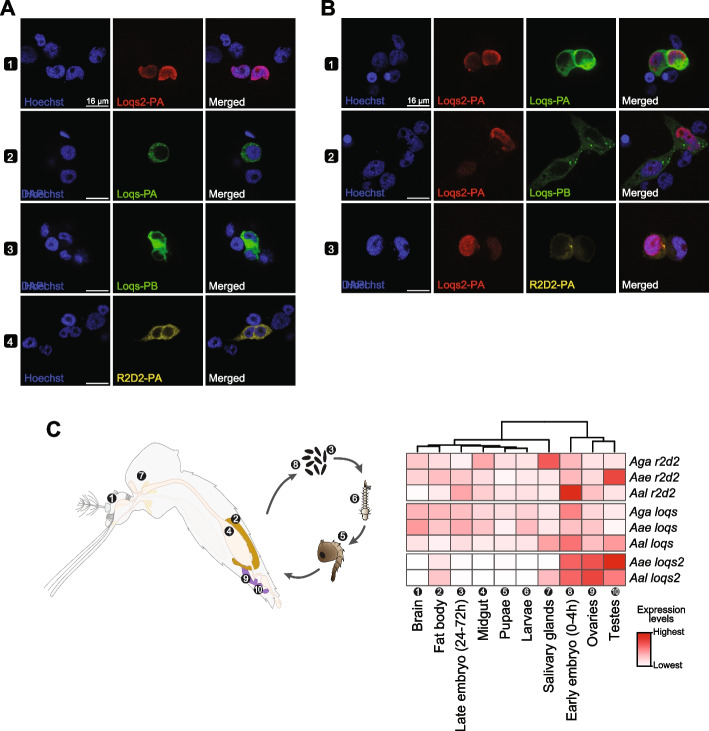
*loqs2* expression and subcellular protein localization differs from *loqs* and *r2d2*. **A-B** Subcellular localization of Loqs2-PA, Loqs-PA, Loqs-PB and R2D2 during overexpression driven by the *polyubiquitin* promoter. Plasmids were transfected individually (**A)** or co-transfected (**B**) in *Ae.* *aegypti* Aag2 cells clone AF5. **C** Heatmap showing the tissue-specific expression of *loqs, loqs2* and *r2d2* among *Ae. aegypti, Ae. albopictus* and *An. gambiae*. The accompanying illustration shows the tissues and developmental stages analyzed. Associated numbers correspond to numbers in the heatmap. Gene expression between tissues was used for hierarchical clustering. White boxes are indicative of no detected mRNA expression

Eukaryotic cells are divided into morphologically distinct compartments. The subcellular localization of a protein influences the accessibility to interact with molecular partners and often gives functional clues of how novel proteins operate. Since *loqs2* originated from a duplication of the first dsRBD of *loqs*, we inquired if both proteins shared the same subcellular localization. For this, we generated plasmids carrying the isoforms *loqs2-RA*, *loqs-RA/-RB* and *r2d2-RA* fused to different immunogenic tags, all under control of the promoter *polyubiquitin* (*PUb*) for expression in *Ae. aegypti* Aag2 cells clone AF5 [46]. Strikingly, we observed that these proteins are localized in different cellular compartments when expressed individually, being Loqs2-PA nuclear while both Loqs isoforms (-PA and -PB) and R2D2-PA are well extended among the cell cytoplasm (Fig. 4A). This raised the question if the cellular localization of Loqs2 could be influenced by Loqs or R2D2, both expected to be present at physiologic levels in *loqs2* transfected cells. We further co-transfected plasmids carrying *loqs2* and each *loqs* or *r2d2* cassettes under the same *PUb* promoter and observed no changes in the localization patterns of Loqs2, Loqs or R2D2 (Fig. 4B). Of note, our group has previously shown that Loqs2 interacts with both Loqs and R2D2 in Aag2 cells [19]. These interactions could appear from shuttling of Loqs and R2D2 to the nucleus or shuttling of Loqs2 to the cytoplasm, but more experiments are necessary to understand these interactions in detail.

Apart from differences in cellular localization, we wondered if *loqs2* would have also evolved an alternative expression pattern compared to *loqs* and *r2d2.* Thus, we used publicly available RNA-seq data to analyze the expression levels of *loqs*, *loqs2*, and *r2d2* in different tissues and developmental stages of *Ae. aegypti* and *Ae. albopictus* (Additional file 2: Table S1) [47–60]*.* We also included expression data from *An. gambiae* to evaluate changes in expression of *loqs* or *r2d2* after the origin of *loqs2* [61–70]*.* We observed that the expression of *loqs2* in both *Ae. aegypti* and *Ae. albopictus* was mostly restricted to reproductive tissues (Fig. 4C), supporting an “out-of-testes” hypothesis of origin [71, 72]. This hypothesis suggests that testes are catalyst of new genes before evolving functions in other tissues. In contrast, *loqs* and *r2d2* showed ubiquitous expression in all three mosquito species (Fig. 4C). In addition to reproductive tissues, we also noted high expression of *loqs2* in early embryos (0-4 h) followed by a sudden decrease in later stages (24-72 h) suggesting that *loqs2* mRNA is maternally deposited in the embryo. In addition, we observed no expression of *loqs2* in larval or pupal stages, revealing that *loqs2* expression is down regulated during mosquito development. It is tempting to speculate that the presence of Loqs2 could impact viral tropism since it is absent from tissues well known to host strong arbovirus replication such as the midgut [73]. Overall, the particular expression pattern of *loqs2* combined with its distinctive subcellular localization gives further support for a hypothesis of evolution by neofunctionalization. Interestingly, the probable “out of testes” origin of *loqs2* allow us to speculate that this dsRBP might have arisen in testes and evolved additional specific functions in the ovary and embryo that might regulate mosquito development.

### *loqs2* acts beyond the miRNA, piRNA and siRNA pathways in the cell

**Fig. 5:**
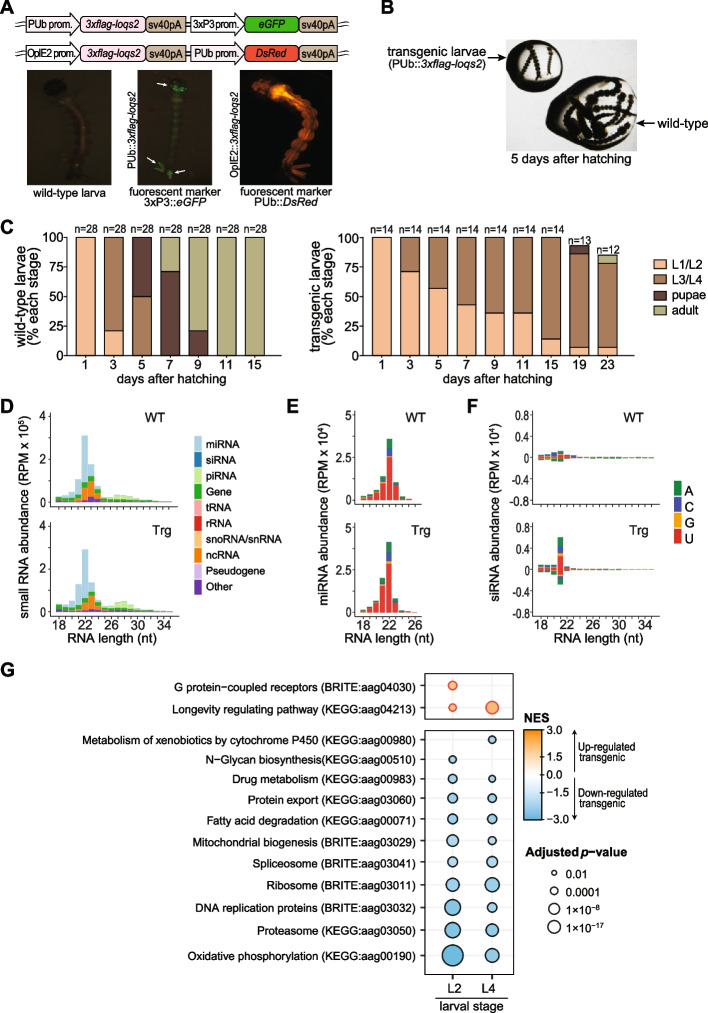
Ectopic expression of *loqs2* in larval stages results in larval growth arrest. **A** Cassettes for ectopic expression of *loqs2* under the control of the *Ae. aegypti polyubiquitin* (*PUb*) promoter or the baculovirus promoter OpIE2. Fluorescent markers under control of the promoters 3x*P3* or *PUb* were used to drive eGFP or DsRed2 expression as transgenesis markers as indicated. Images are representative of larvae from each transgenic line. **B** Wild-type (non-transgenic) sibling larvae, reared at the same water container, showed normal development until pupal stage while transgenic larvae exhibited developmental arrest at L2-L3 stages of development at day 5 post-hatching. **C** Stacked bar plots comparing development of wild-type and transgenic mosquitoes ectopically expressing *loqs2* under control of the baculovirus promoter OpIE2. **D–F** sRNA sequencing analyses of larvae L2 ectopically expressing *loqs2* compared to wild-type siblings. **D** sRNA abundance (18 to 35 nt) from wild-type (WT) and transgenic (Trg) larvae L2 colored by genomic origin. **E** sRNA abundance (18 to 26 nt) that mapped to a curated miRNA reference [76]. **F** sRNA abundance (18 to 35 nt) that mapped to a de novo generated siRNA cluster reference. Bar colors in **E** and **F** represent the first 5' nucleotide base distribution. sRNA abundances in **D–F** are reported as reads per million mapped (RPM). **G** Gene set enrichment analysis (GSEA) of larvae ectopically expressing *loqs2* compared to wild-type siblings showed a major transcriptional shutdown on the transcription of metabolic pathways in transgenic individuals

To investigate potential roles of *loqs2*, we overexpressed this gene during larval development in *Ae. aegypti* and analyzed the abundance of small RNAs and mRNA transcription levels*.* For this, we generated two independent transgenic mosquito lines expressing *loqs2* under the control of either the *PUb* gene promoter or the baculovirus promoter OpIE2 (Fig. 5A; Additional file 1: Fig. S5A). These promoters drive ubiquitous gene expression at late larval stages of *Aedes* and, in both cases, caused delayed larval development compared to non-transgenic siblings. Most transgenic individuals showed retarded molting with consequent prolonged L3 and L4 larval stages (Fig. 5B, C). Of note, the observed delay in molting is not related to the expression of fluorescent proteins since they do not impact larval development [74, 75].

To understand if *loqs2* overexpression would affect the small RNA abundance, we compared transgenic larvae and wild-type siblings at the L2 stage (Fig. 5D; Additional file 1: Fig. S5B). We observed that RNAs with length between 18 and 35 nucleotides (nt) mapping to different genomic features (*i.e.*, miRNAs, protein coding genes, piRNA clusters, etc.) showed no significant differences in abundance or length distribution. We further dissected sequences matching mature miRNAs or siRNA clusters, since their biogenesis depends on Loqs in *Ae. aegypti* [14]. We also included piRNA clusters, which are transcribed by the piRNA pathway to prevent transposable element (TE) propagation [77]. We observed no differences in miRNA abundance or frequency of first 5' nucleotide between transgenic or wild-type larvae (Fig. 5E; Additional file 1: Fig. S5C). In a similar fashion, no significant differences in the abundance of piRNAs (Additional file 1: Fig. S6A, B) or in the expression of TE families (Additional file 1: Fig. S6C) were observed when comparing transgenic to wild-type control larvae. On the other hand, we observed a subtle increase in the abundance of siRNAs with 21 nt length in transgenic larvae compared to wild type (Fig. 5F; Additional file 1: Fig. S5D). Such increase in siRNA levels has also been observed during overexpression of *Dicer-2* and *r2d2* in the midgut of *Ae. aegypti* mosquitoes and indicates increased activity of the siRNA pathway [78]. Interestingly, only a few genomic loci were responsible for the difference in siRNA abundance we observed in larvae, possibly as result from Loqs2 controlling the biogenesis of siRNAs from these genomic loci in cells ectopically expressing this protein. Overall, our data suggest that *loqs2* overexpression positively impacts the siRNA biogenesis via RNAi without affecting the piRNA and miRNA pathways, the latter fully dependent on Loqs.

We further evaluated the transcriptome of transgenic larvae to inquire the cause of developmental arrest after *loqs2* overexpression. Transcriptomic analysis of transgenic larvae and wild-type siblings at L2 and L4 stages showed significant differences in gene expression between both conditions. In transgenic mosquitoes, gene-set enrichment analysis (GSEA) showed a significant decrease in expression of genes related to protein synthesis and degradation, genetic information processing and energetic metabolism. Notably, this analysis also showed a significant upregulation of biological processes related to signal transduction via G-protein coupled receptors and the longevity regulating pathway. (Fig. 5G; Additional file 1: Fig. S7). These results suggest that the developmental arrest at larval stages could be associated with a broad metabolic suppression that ultimately leads to cellular stress. The mechanism where Loqs2 affects transcription seems independent of the miRNA-guided control of gene expression, since no significant change in miRNA abundance has been observed in transgenic larvae. Altogether, our results show evidence of *loqs2* neofunctionalization, playing a different role than its precursor *loqs* and acting beyond the siRNA, piRNA and miRNA pathways in the cell.

## Discussion

Our group recently reported the identification of *loqs2*, a dsRBP that enables the control of DENV and ZIKV in *Ae. aegypti* mosquitoes, suggesting that *loqs2* could have evolved to be a regulator of the mosquito antiviral defense [19]. In the present study we investigated the origin and evolution of *loqs2* and used genetic tools to shed some light on its biological role. We found that *loqs2* is a paralog of *loqs* that originated around 31 MYA in an ancestor of the *Aedes Stegomya* subgenus and evolved by neofunctionalization driven by positive selection. Remarkably, the origin of *loqs2* appear to be an exception to the general model of dsRBP origin [31]. While most dsRBPs are result of gene duplications, our findings suggest *loqs2* is the product of two independent duplications of the first dsRBD of *loqs* (*loqs*-D1) that inserted into a non-genic DNA region that further evolved to become *loqs2*. This unexpected origin certainly provided ground for the evolution of new functions for *loqs2* since little amino acid sequence conservation, apart from its functional domains, exists between Loqs2 and its predecessor Loqs. We showed evidence for neofunctionalization resulting from positive selective forces acting on the gene. Relaxed purifying selection may have facilitated the accumulation of mutations while positive selection may have driven adaptive innovations, ultimately leading *loqs2* towards new functions. The finding that *loqs2* is still evolving fast, as showed by the Ka/Ks ratio, suggest *loqs2* is still diversifying and probably still fine-tuning its functions. These results combined with the high conservation among the amino acids predicted to be involved in dsRNA binding allow us to speculate that *loqs2* evolved new functions while maintaining its RNA interaction capabilities.

Our combined evidence about the origin and evolution of *loqs2* (*i.e.*, mode of origin, evolution, subcellular localization, and tissue-specific expression) support an “out of testes” origin [71, 72]. Under this hypothesis, we speculate that *loqs2* initially evolved as a testes-specific gene, favored by the intrinsic properties of this tissue (*e.g.*, promiscuous transcription, fast evolution) [79, 80]. Subsequently, *loqs2* would have gained the regulatory elements and functions to become a stable coding gene that eventually expanded to other tissues. Such tissue-specific regulation in gene expression certainly influenced the molecular evolution of *loqs2*. Our findings here, combined with previous results from our group, drive us to hypothesize that *loqs2* may have originally evolved germline specific functions.

In line with our Loqs2 neofunctionalization hypothesis*,* our functional analyses revealed that *loqs2* expression is halted during embryogenesis and restored only in the adult stage of the mosquito. We detected the *loqs2* mRNA in the first hours of embryogenesis (0 to 4 h) that abruptly disappears later, suggesting that *loqs2* mRNA is maternally deposited during the early phase of egg development and cleared at later stages of embryogenesis [81]. An altered state where *loqs2* was ectopically expressed in the larval stages caused a strong developmental delay that impaired the transition between larval stages (Fig. 5C). *loqs2* ectopic expression during larval stages caused a broad suppression of metabolic pathways that did not correlate with changes in small RNA abundances, as would typically be expected for a protein directly involved in the RNAi pathway. Altogether, these data suggest that *loqs2* unexpected origin provided the conditions for the evolution of new functions not associated to the RNAi pathway. This study forms the basis for more detailed exploration of the role of *loqs2* in *Aedes* mosquitoes, where its function seems to be both tissue- and development-specific. Further experiments are necessary to investigate its context-specific roles in a mechanistic manner.

Our data does not completely rule out that there was some degree of subfunctionalization since *loqs* might have had alternative functions that were attributed to *loqs2* after its origin. In *D. melanogaster,* Loqs-PA interacts with Dicer-1 exclusively through its third dsRBD, while Loqs-PD interacts with Dcr-2 through a 37 aa C-terminal region [82–84]. These regions are not conserved in *loqs2*, indicating a different site or a different mechanism for interaction with protein partners, which also leaves room to speculate that the interaction between Loqs2 and partners such as R2D2 and Loqs in *Ae. aegypti* might be indirect and dependent on binding to dsRNA [19]. In *D. melanogaster,* both dsRBDs of Loqs seem to be equally capable of binding to dsRNA [32], but the first dsRBD of *D. melanogaster* Loqs-PD is essential for the cleavage of suboptimal dsRNA substrates by Dicer-2 in vitro [84]. The flexibility and length of the amino acid linker connecting both dsRBDs in *D. melanogaster* Loqs seems to allow independent binding of each domain to a dsRNA substrate, with consequent and mobile reversible interaction [32]. In *Aedes* mosquitoes, Loqs has a linker length between dsRBDs D1 and D2 of 29 amino acids while this distance in Loqs2 is only of 15 amino acids. We speculate that the short linker between the two dsRBDs of Loqs2 results in lower flexibility, possibly causing a shift in binding affinities to dsRNA substrates. Despite a dual role of the *Ae. aegypti* Loqs into the miRNA and siRNA pathways, our small RNA sequencing data showed that ectopic expression of *loqs2* in larvae only caused a minor impact on the siRNA biogenesis, suggesting that *loqs2* might have evolved to deal with different dsRNA substrates from *loqs*. Also, it is tempting to think that the nature of Loqs2, as a small dsRBP that probably acts mainly as a sensor of dsRNA, allowed for the evolution of functional pleiotropy determined by the tissue-specific protein partners*.* This hypothesis might help explaining the varied metabolic pathways regulated as a response to the ectopic expression of *loqs2* in our transgenic larvae and explain the previously observed antiviral effect in the midgut of mosquitoes [19].

## Conclusions

In summary, our work shed light into the origin, evolution and function of *loqs2*, a double-stranded binding protein found specifically in the *Stegomya* subgenus of *Aedes* mosquitoes. We show evidence of neofunctionalization of *loqs2* driven by positive selection and relaxed purifying selection compared to it parental gene *loqs*. Our data show that *loqs2* has tissue- and development-specific expression with potential regulatory functions that extend beyond the RNAi pathway. Interestingly, this novel dsRBP may have played a role in shaping the capacity of *Aedes* mosquitoes as arbovirus' vectors. With the recent advances in genetic-based strategies, *loqs2* seems like a promising target for genetic interventions since regulation of this *Aedes* specific gene could render mosquitoes resistant to virus infection while also strongly affecting its larval development.

## Methods

### *loqs* and *loqs2* sequence retrieval from publicly available high-throughput sequencing data of different species and exome-seq alignment

Public high-throughput RNA-seq and DNA-seq libraries were obtained from the NCBI/SRA database (Additional file 2: Table S1). The pipeline nf-core/rnaseq v3.12.0 [85] was used to analyze the RNA-seq libraries from different mosquitoes. Briefly, raw reads were preprocessed for removal of low quality bases and adaptor sequences using cutadapt v3.7 [86] and aligned to the *Ae. aegypti* and *Ae. albopictus* reference genomes (Vectorbase release 63) [20] using STAR v2.7.10a [87]. To overcome the expected divergence between the RNA-seq data and the reference genomes, we set the alignment type to “local” and allowed a maximum mismatch percentage of 50% of the read size. Whole genome sequencing (WGS) of *Ae. mascarensis* and *Ae. bromeliae* and exome sequencing of *Ae. aegypti* individuals from a Senegal forest population were preprocessed as above using trimmomatic v0.39 [88] and overlapping reads were merged with FLASH v1.2.11 [89]. Both paired- and single-end pseudo-reads were mapped to the *Ae. aegypti* and *Ae. albopictus* reference genomes using BWA-mem2 v.2.0pre2 [90]. Alignment files from RNA-seq and DNA-seq data were sorted and merged when necessary with SAMtools v1.17 [91]. Read duplicates were marked with Picard v2.21.5 [92]. The RNA-seq and WGS alignments to the regions comprising *loqs* and *loqs2* were manually inspected using JBrowse2 v2.6.2 [93] and unique mappers were exported as SVGs and plotted with Inkscape v1.3 (Inkscape Project, 2023). The cladogram summarizing the alignment results was plotted based on the phylogenetic relationships reported in [21]. WGS and exome-seq alignments were used for population genomics.

### Synteny and exon–intron structure analyses

Synteny analysis of the *loqs2* genomic vicinity was plotted according to the VEuPathDB orthology maps [20]. We used the reference genomes of *Ae. aegypti, Culex quinquefasciatus, An. gambiae* and *G. palpalis* VEuPathDB release 63 for the comparative analysis. For exon–intron structure analyses the references genomes and genome annotations of *Ae. aegypti* and *Ae. albopictus* were analyzed using JBrowse2 v2.6.2 [93]. Plots were generated using Adobe Illustrator 2024.

### Transcriptomic analyses of tissue-specific libraries

To quantify the expression profiles of *loqs, loqs2* and *r2d2* among different tissues from *Ae. aegypti, Ae. albopictus* and *An. gambiae* mosquitoes, public RNA-seq libraries were obtained from NCBI/SRA (Additional file 2: Table S1). Reads were mapped to the decoy-aware reference transcriptome of each species (Vectorbase release 52) using Salmon v1.5.2 [94]. Salmon quantifications were imported into R v4.3.0 [95] using tximport v1.28.0 and data normalization was performed using the packages EdgeR v3.42.4 and TMM [96–98]. The heatmap was generated using the R package gplot v3.1.3.

### Phylogenetic analyses

For phylogenetic analyses we used a set of mosquitoes (*Ae. aegypti, Ae. albopictus, C. quinquefasciatus, An. gambiae* and *An. coluzzii*) and fly (*L. longipalpis, S. calcitrans* and *G. fuscipes*) species to infer the evolutionary history of *loqs2* at three different evolutionary ranges: *(i)* the dsRBP orthologs, *(ii)* the dsRBDs from the dsRBPs in strategy *i* and, *(iii)* the closely related evolutionary history of *loqs2* dsRBDs. Input sequences were aligned using either PRANK_+F_ (strategies *i* and *ii)* [99] or MACSE v2.06 (strategy *iii)* [100], and the substitution model and phylogenetic relationships were inferred using IQ-tree2 [101]. For all cases, the best fit model was determined based on the Bayesian information criterion (BIC) and the maximum-likelihood consensus tree was generated with 1000 ultra-fast bootstraps iterations. All trees were rooted at the midpoint using Figtree v1.4.4 [102] for visualization purposes. For the dsRBP tree, the amino acid sequences were retrieved from VeuPathDB [20] using the Double-stranded RNA-binding domain InterPRO accession (IPR014720), including Dicer-2. The WAG amino acid exchange matrix under FreeRate heterogeneity model with 5 categories (WAG + R5) was determined to be the best fit model. For the dsRBD tree, we used the ScanProsite tool to retrieve the dsRBD amino acid sequences from the dsRBPs identified in strategy *i.* The Q.insect amino acid exchange matrix [103]with discrete Gamma model with 4 rate categories (Q.insect + G4) was determined to be the best fit model based on the BIC. For the *loqs2* close evolutionary history, we build the tree using the coding sequences of each domain from *loqs2,* and the first dsBRDs of *loqs* and *r2d2* from *Ae. aegypti* and *Ae. albopictus*. The KOSI07 empirical codon model with amino-acid frequencies given by the protein matrix under FreeRate heterogeneity model with 3 categories (KOSI07 + FU + R3) was determined to be the best fit model.

### Loqs and Loqs2 dsRBDs tridimensional modeling and structural and amino acid sequence pairwise comparisons

Homology models of the dsRBDs of *Ae. aegypti* and *Ae. albopictus* Loqs and Loqs2 were built using a template-based method. The *D. melanogaster* Loqs-PD dsRBD1 structure (PDB id: 5NPG) was used as the structural template to model the dsRBD1 of Loqs and the dsRBDs of Loqs2. For Loqs dsRBD2 models, the *D. melanogaster* Loqs-PD dsRBD2 structure (PDB id: 5NPA) was used as template [32]. Models were made with MODELLER v9.24 [104] using the automodel class. In each case, 100 models were produced, and the model with lowest MODELLER DOPE score was selected. Quality of each selected model was checked by the analysis of its Ramachandran plot built with PROCHECK [105]. All selected models presented at least 88% of their residues located in the most favored regions of the plot and no more than 1 residue located in a disallowed region. Model visualization was done using pymol v2.4.0 [106]. For structural comparisons, the modeled *Ae. aegypti* and *Ae. albopictus* Loqs and Loqs2 dsRBDs were compared to each other using the “All against all” structure comparison of DALI server [33]. In brief, DALI calculates a matrix of pairwise structural similarities and reports a dendrogram derived from the average linkage clustering of the matrix. For the amino acid sequence global alignment and pairwise comparisons, the sequences of *Ae. aegypti* and *Ae. albopictus* Loqs and Loqs2 dsRBDs were pairwise aligned with EMBOSS Needle under the BLOSUM62 substitution matrix [107]. The reported metrics were used to build a matrix of identity percentages and a matrix of global alignment percentages (calculated as 100% minus the gap percentage) that were summarized in a correlation matrix plotted using R v4.3.0 and the package ggplot2 v3.4.2. The reported dendrogram was built from the matrix of identity percentages using the hclust function from R v4.3.0 and the package ape v5.7–1 [108].

### Evolutionary analyses using a phylogenetic framework

We used the phylogenetic tree from strategy *iii* (see above) as evolutionary hypothesis to perform three tests of selection based on the rates of nonsynonymous and synonymous substitutions (*ω*). A test for relaxation or intensification of the strength of natural selection of *loqs2* dsRBDs compared to *loqs* dsRBD1 was performed with RELAX [37] implemented in the datamonkey server [109]. RELAX uses the parameter *k* to test for relaxed (*k* < 1*)* or intensified (*k* > 1) selection between a set of test branches compared to a set of reference branches. RELAX tests for statistical significance (*p-value* < 0.05*)* by comparing a null model (*k* is fixed to 1) to an alternative model (*k* is a variable parameter) using a likelihood ratio test (LRT). Paired comparisons were made between the *Aedes loqs2* dsRBD1 or dsRBD2 terminals as test branches and *loqs-D1* terminals as reference branches. To test for positive selection at *loqs2* dsRBDs we employed the branch-site-model implemented in abSREL [40] within datamonkey server. This method estimates the *ω* rates at branch- and site-level of the test branch, infers the optimal number of *ω* classes, and tests if a proportion of sites have evolved under positive selection. abSREL test for positive selection by using a LRT to compare the fitted adaptive model to a null model that only allows neutral and negative selection. We used this methodology to test for episodic positive selection along the branches of *loqs2-*D1 and *loqs2-*D2 before the bifurcation of *Aedes aegypti* and *Ae. albopictus*. Given that our tree is not monophyletic for *loqs-D1,* we conducted the absREL tests for each *loqs-*D1 branch individually. To evaluate the evolution of these branches at the site-level we used FEL (Fixed Effects Likelihood) [110], implemented in the datamonkey server.. This approach estimates the site-specific nonsynonymous (dN) and synonymous (dS) substitution rates using a maximal likelihood approach. In summary, FEL uses the entire dataset to optimize branch lengths and nucleotide substitution parameters, then fits a MG94xREV model to each codon site and infers site-specific dN and dS substitution rates. Finally, it fits a neutral and selection model to every codon and calculates a standard LRT to decide if the site is significantly evolving non-neutrally.

### Evolutionary analyses using population genomics

Public exome data of an *Ae. aegypti* population from a Senegal forest area [23] (Additional file 2: Table S1) were mapped to the reference genome of *Ae. aegypti* (described above) and variant calling was performed with GATK v4.1.4.1 [111] with a heterozygosity prior of 0.003125 as reported by Redmond et al. [27] for *Ae. aegypti.* SNP calling was performed only for *loqs, loqs2, Dicer1* and *Dicer2* genomic loci including flanks of 2-Mb. Resultant SNP data was hard filtered with VCFtools v0.1.17 and indels were removed [112]. To determine the derived alleles, we performed the same SNP calling pipeline for *Aedes mascarensis* whole genome sequencing data (Additional file 2: Table S1). Finally, we used bcftools and GATK v4.1.4.1 to transform the SNP variants into a FastA sequence and used a custom python script to create a multi-fasta file for *loqs* or *loqs2* coding sequences. These files were used to calculate the nonsynonymous (*Ka*) to synonymous (*Ks*) substitutions using the iMKT web server [42].

### Plasmid construction

Plasmids were built using both GoldenGate and Hi-Fi cloning [113, 114]. Sequences and annotations are provided in GenBank format on the Additional file 3. Briefly, DNA fragments were PCR-amplified using Phusion High-Fidelity DNA polymerase (Thermo Scientific). Primers used in PCR reactions for GoldenGate were designed to add BsaI sites in both extremities of the amplicon while the primers for HiFi cloning were designed to include a complementary overhang in their extremities [114]. Primer sequences and construction schemes for each plasmid are available from the corresponding authors, RPO and JTM, upon reasonable request. All PCR products were cloned into a modified pBluescriptII KS( +) (Addgene #62540) lacking BsaI restriction site in a one-step digestion/ligation using FastDigest SmaI or FastDigest EcoRV (Thermo Scientific) and T4 DNA-ligase (Invitrogen) with 10 mM ATP. Final plasmid assembly by GoldenGate cloning was conducted as described previously [114]. Successful cloning was confirmed by Sanger sequencing (GATC Biotech).

### Cell culture and plasmid transfection in Aag2 cells

*Ae. aegypti* Aag2 cells (clone AF5) were grown at 25 °C in L15 medium supplemented with 10% FBS, 1% penicillin–streptomycin and 1% GlutaMAX (Gibco). Before transfection, 5 × 10^5^ cells were seeded per well of 24-well plates and transfected with 400 ng of plasmid DNA using Effectene Reagent (Qiagen), following the manufacturer’s instructions. Medium was replaced 18 h after transfection and cells were allowed to grow for 3 days. After this period, cells were mechanically detached and directly transferred to a Millicell EZ SLIDE 8-well glass slides (Merck Millipore) and cultivated for 24 h prior to further usage.

### Indirect immunofluorescence assay

Cells were gently washed once with ice-cold PBS solution (13 mM NaCl, 0.7 mM Na_2_HPO_4_, 1 mM NaH_2_PO_4_ at pH 7.2) and immediately fixed using 4% paraformaldehyde for 30 min at 25 °C. After, each well was washed twice with PBS for 5 min and incubated for 1 h in blocking solution PBST (1 × PBS + 1% BSA + 0.1% Triton X-100) with gentle rocking at 25 °C. Proteins fused to the V5 or FLAG epitope tags were detected using the mouse monoclonal antibody anti-V5 (Invitrogen, catalog R960-25) or the rabbit polyclonal antibody anti-DDDDK (anti-FLAG, Abcam, catalog ab1162). Both antibodies were diluted 1:200 in PBST and incubated for 1 h at room temperature. Cells were washed 3 times for 5 min each in PBST and incubated for 1 h at room temperature with goat anti-mouse IgG antibody conjugated to Alexa 568 (Molecular Probes, 1:400), goat anti-rabbit antibody conjugated to Alexa 647 (Molecular Probes, 1:400) and Hoechst 33342 (trihydrochloride, trihydrate, Molecular Probes, 1:1,000). Subsequently, wells were washed twice with PBST, rinsed with PBS and placed onto Vectashield Antifade Mounting Medium (Vector laboratories). Images were obtained with an Apotome.2 microscope (Zeiss).

### Mosquito transgenesis and developmental assays

Embryo microinjection was performed with small modifications, as previously described [19]. Briefly, *Ae. aegypti* mosquitoes (Bangkok background) were allowed to blood feed in an anesthetized mouse and blood fed females were kept for 4 days in a cage containing 10% sucrose ad libitum. Mosquitoes were transferred to an small 50 mL cup containing a moist filter paper folded in a conical shape. Freshly laid eggs (25 ~ 45 min) were aligned in parallel against the internal side of a U-shaped nitrocellulose membrane in contact with an overlaying filter paper soaked in demineralized water. Aligned eggs rested in a humid chamber for 30–60 min after alignment, until the eggs turned dark grey. Mixes containing 100 ng/μl of a *piggyBac* transposase helper-plasmid and 400 ng/μl of either plasmid carrying *piggyBac*-flanked cassettes (Fig. 4B) were diluted in 0.5 × PBS and injected at the embryo posterior pole under a Nikon Eclipse TE2000-S inverted microscope, using a Femtojet injector (Eppendorf) and a TransferMan NK2 micromanipulator. Before being allowed to dry, microinjected eggs were kept for 2 days diagonally in a container with 1-cm-deep water. The filter paper had direct contact with water, which kept the eggs moist by capillarity [114]. Surviving eggs were hatched under vacuum and larvae showing constitutive expression of fluorescent markers were kept for further crossing with wild-type individuals. Larvae obtained at the next generation were screened using fluorescence to track for transgene integration on their genomes. Wild-type and transgenic larvae were bred in the same container and same conditions. Individuals were accounted daily for development and survival for a total of 23 days.

### mRNA and sRNA high-throughput RNA-seq library construction and sequencing

Total RNA from 5 to 10 individual larvae were extracted using TRIzol (Invitrogen) following the recommended protocol. RNA integrity was verified using the 2100 Bioanalyzer system (Agilent). mRNA libraries were constructed using the kits NEBNext Poly(A) mRNA Magnetic Isolation Module and NEBNext UltraTM II Directional RNA Library Prep Kit for Illumina (New England BioLabs) following the manufacturer protocol. Small RNA libraries were built using the kit NEBNext Multiplex Small RNA Library Prep (New England BioLabs) following the manufacturer protocol, except for the 5’ RNA adaptor that was modified to include 6 nt at its 5’ extremity. These extra nucleotides are sequenced along with the small RNA ligated and removed at the bioinformatic analysis. Indexed libraries were pooled and sequenced at the GenomEast sequencing platform at the Institut de Génétique et de Biologie Moléculaire et Cellulaire of Strasbourg, France.

### mRNA transcriptomic analyses

We used the pipeline nf-core/rnaseq v3.12.0 [85] to analyze the mRNA-seq libraries. Raw sequenced reads from mRNA libraries with an average quality score above phred 25 had adaptors removed using Trimmomatic v0.39 [88]. Processed reads were aligned to the *Ae. Aegypti* reference genome (Vectorbase release 63) using STAR v2.7.10a [87] and alignments were quantified with Salmon v1.10.1 [94]. Salmon quantifications were imported into R v4.3.0 (R Core Team 2021) using tximport v1.28.0 [98]. Data filtering and normalization were performed using the packages EdgeR v3.28.1 and TMM [96, 97]. Ranked lists of gene expression for each comparison (transgenic against wild-type larvae) were used as input for Gene Set Enrichment Analysis (GSEA) [115] using the R package fgsea v1.12.0 [116] and gene-sets comprising KEGG pathways and BRITE hierarchies. Sets with adjusted *p*-value < 0.01 were considered in our analysis. To reduce redundancy, dependent gene-sets were collapsed using the “collapsePathways” function of fgsea with a pval.threshold of 0.01.

### sRNA transcriptomic analyses

Raw sequenced reads from small RNA libraries were preprocessed by trimming of the added 6 nt at the 5’ extremity and adaptor removal using cutadapt v3.7 [86]. Low Phred quality (< 20), ambiguous nucleotides and/or with length shorter than 15 nt were also discarded. For the prediction of siRNA and piRNA clusters, we partitioned the reference genome of *Ae. aegypti* (Vectorbase release 63) [62] into 2 kb segments. The processed reads were then aligned to this segmented genome, allowing for 2 mismatches and up to 10 valid alignments with Bowtie v1.2.3 [117]. Alignment files were merged and clusters were predicted using in-house Perl v5.16.3, BioPerl library v1.6.924 scripts [118]. In brief, the script classifies the 2 kb segments based on the distribution of read counts across different size ranges (*i.e.*, siRNA = 20–22 nt; piRNAs = 24–29 nt; miRNA = 22–24 nt; other = 15–19 nt) to determine the most likely type of sRNA cluster. Following the cluster prediction, we remapped the processed reads to the siRNA or piRNA cluster reference, allowing for 1 mismatch. Counts were normalized by RPM and plotted using Perl v5.16.3, Python v3.10.9, and R v4.3.0 scripts with the libraries BioPerl library v1.6.924, pandas v2.0.2, reshape v0.8.9, ggplot2 v3.4.2, ComplexHeatmap v2.16.0 and circlize v0.4.15 [118–123]. As for the miRNAs, we used a curated miRNA reference [76] for mapping, and counts were normalized and plotted as described above. To analyze the overall distribution of small RNAs, the processed reads were mapped to the reference genome of *Ae. aegypti* with Bowtie v1.2.3, allowing only for unique mappers with up to 1 mismatch. We utilized Perl v5.16.3, BioPerl library v1.6.924 and R v4.3.0 scripts to count the reads, normalize by RPM, and plot their size distribution based on their base type or genomic features [118]. Regarding the distribution by genomic feature, we prepared a set of genomes containing a single class of genomic loci (*e.g.*, ncRNA, rRNA, protein coding gene) using AGAT v1.2.0. Fastq files containing the reads mapping to the reference genome were prepared and remapped sequentially to the sRNA references and the tailored genomes. This sequential remapping was designed to retain only unique mappings while the unmapped or non-uniquely-mapped reads were passed on to the next mapping round.

### Supplementary Information

**Additional file 1: Fig. S1. **(Related to Fig. 1). Non-collapsed phylogenetic tree from Fig. 1A and read mapping of RNA-seq and whole-genome-seq data from different *Aedini* species aligned to the *Ae. aegypti* and *Ae. albopictus* reference genomes. **A** Terminals correspond to the VEuPathDB accession numbers. The dsRBP orthology groups are indicated by black bars and the protein lengths of the *Ae. Aegypti* orthologs are indicated in parentheses. Tree was rooted at the midpoint for visualization purposes. Node values correspond to the percentages of 1000 ultra-fast bootstrap iterations**.** Branch lengths represent substitutions per site. **B** *loqs* and *loqs2* exons are colored in purple and blue respectively. Black boxes represent the read mapping along *loqs* and *loqs2* gene sequences. Lines represent intronic regions of both gene sequences and reads. *indicates species where whole genome sequencing were analyzed. **Fig. S2. **(Related to Fig. 2). Non-collapsed phylogenetic tree from Fig. 2B. Terminals correspond to the VEuPathDB accession numbers. Each orthology group’s dsRBDs are numbered and indicated by black bars. The tree was rooted at the midpoint for visualization purposes. Node values correspond to the percentages of 1000 ultra-fast bootstrap iterations**.** Branch lengths represent substitutions per site. **Fig. S3. **(Related to Fig. 2). Cladogram built from the Loqs-D1-Loqs2 subtree from Fig. 2B and exon–intron organization of the *loqs* and *loqs2* genes in *Ae. aegypti* and *Ae. albopictus.* **A** The cladogram was built with equal branch lengths. The clade containing the Loqs2 dsRBDs is colored in grey. **B** The exon–intron structure of the *loqs* and *loqs2* genes is indicated by boxes (exons) and continuous lines (introns). dsRBD regions are indicated in blue boxes. Exons are represented by boxes and introns by grey continuous lines. **C** Zoomed-in view of the amino-acid sequences from the exon–intron-exon boundaries within the dsRBD domains of *loqs* and *loqs2*. **Fig. S4. **(Related to Fig. 3). dsRBDs from Loqs2 present high conservation among the residues important for folding and dsRNA binding. Translated amino acid alignment from the codon-based alignment used to build phylogenetic framework reported in Fig. 3B. The top row displays the consensus residues for dsRBD folding and dsRNA binding, as previously reported  [39]. Conserved residues along the alignment are highlighted in pale green boxes. The specific residues necessary for dsRNA binding are highlighted in dark green. Secondary structure elements of the dsRBDs are showed at the bottom of the alignment. Sites with significant evidence of purifying or diversifying selection, as detected by FEL analysis, are indicated by blue or red boxes, respectively. **Fig. S5.** (Related to Fig. 5).** A** Expression levels of *loqs2* in transgenic or wild-type sibling larvae quantified by RT-qPCR. **B** sRNA abundance (18 to 35 nt) from wild-type and transgenic larvae (related to Fig. 5D). **C-D** Heatmaps showing the abundances of sRNAs mapped to the miRNAs and siRNA clusters references among the wild-type and transgenic larvae (related to Fig. 5E-F). Abundances are reported as reads per million mapped (RPM) and bar colors represent the nucleotide base distribution. Only normalized abundances > 50 RPM are reported. Normalized mapped sRNA abundances were used for hierarchical clustering*.* Names of the siRNA clusters are given by the genome version, followed by the chromosome and initial genomic position of the cluster. **Fig. S6. **(Related to Fig. 5). sRNA abundance (18 to 35 nt) from wild-type and transgenic larvae that mapped to a de novo generated piRNA cluster reference, and expression of transposable element (TE) families among L2 and L4 wild-type and transgenic larvae. **A** bar colors represent the nucleotide base distribution. **B** Heatmap shows the abundances of sRNAs mapped to the de novo predicted piRNA clusters. Normalized mapped sRNA abundances were used for hierarchical clustering*.* In **A** and **B,** abundances are reported as reads per million mapped (RPM). **C** The heatmap shows the normalized expression of TE families as log2 counts per million (Log_2_CPM). TE family expression between conditions and TE families was used for hierarchical clustering. **Fig. S7. **(Related to Fig. 5). Ectopic expression of *loqs2* in larval stages lead to major metabolic shutdown. GSEA analysis plot showing extended gene sets prior to redundancy removal relative to Fig. 5G.

**Additional file 2: Table S1.** SRA identifiers of the libraries used in this study. **Table S2.** Metrics of divergence and McDonald-Kreitman test (MKT) for *loqs, loqs2, dicer1* and *dicer2* from an *Ae. aegypti* population from a forest in Senegal compared to *Ae. mascarensis.*

**Additional file 3:** Zip compressed file containing plasmid maps in Genbank format. The following files are available: Plasmids used for piggyBac mediated transgenesis in mosquitoes: Files (879 GG PUb-3xFLAG_loqs2_Sv40, 3xP3-eGFP_SV40.gb) and (992 GG OpIE2-3xFLAG_Loqs2_Sv40, PUb-dsRED_Sv40.gb). Plasmids used for transfection in vitro: (993 GG PUb-3xFLAG_Loqs2_Sv40, opIE2-Puro_Sv40.gb), (1000 GG PUb-V5_r2d2_Sv40, opIE2-Puro_Sv40.gb), (1001 GG PUb-V5_LoqsPA_Sv40, opIE2-Puro_Sv40.gb), (1002 GG PUb-V5_LoqsPB_Sv40, opIE2-Puro_Sv40.gb).

## Data Availability

The authors confirm that all relevant data are included in the main manuscript and Additional files provided with the manuscript. Transcriptome libraries from this study have been deposited on the Sequence Read Archive (SRA) at NCBI under project number PRJNA1061509. Other publicly available RNA-seq data sets were obtained from SRA and accession numbers are provided on the Additional file 2: Table S1. Scripts used in this work to perform analyses and generate figures were deposited at GitHub (github.com/CarlosEstevez45/loqs2_evolution). Plasmid maps are available in Genbank format on the Additional file 3.

## Abbreviations

dsRBP: Double-stranded RNA binding protein
loqs: Loquacious
RNAi: RNA interference
Arboviruses: Arthropod-borne viruses
DENV: Dengue virus
ZIKV: Zika virus
CHIKV: Chikungunya virus
siRNA: Small interfering RNA
dsRNA: Double-stranded RNA
Dcr-2: Dicer-2
AGO2: Argonaute-2
RISC: RNA-induced silencing complex
dsRBD: Double-stranded RNA binding domain
MYA: Million years ago
Dcr-1: Dicer-1
dN/dS: Ratio of nonsynonymous to synonymous substitution rates
aBSREL: Adaptive Branch-Site Random Effects Likelihood
Aae: *Aedes aegypti*
Aal: *Aedes albopictus*
Aga: *Anopheles gambiae*
FEL: Fixed Effects Likelihood
MKT: McDonald and Kreitman test
iMKT: Integrative McDonald and Kreitman test
miRNA: MicroRNA
piRNA: Piwi-interacting RNA
PUb: Polyubiquitin
OpIE2: Baculovirus promoter OpIE2
sRNA: Small RNA
TE: Transposable element
GSEA: Gene-set enrichment analysis
WGS: Whole genome sequencing
