# Supplementary material for: Neofunctionalization driven by positive selection led to the retention of the loqs2 gene encoding an Aedes specific dsRNA binding protein
Source: BMC Biol. 2024 Jan 25;22:14. doi: 10.1186/s12915-024-01821-4 (PMC10809485; doi:10.1186/s12915-024-01821-4)
Supplement: Supplementary file 1 — Additional file 1: Fig. S1. (Related to Fig. 1). Non-collapsed phylogenetic tree from Fig. 1A and read mapping of RNA-seq and whole-genome-seq data from different Aedini species aligned to the Ae. aegypti and Ae. albopictus reference genomes. A Terminals correspond to the VEuPathDB accession numbers. The dsRBP orthology groups are indicated by black bars and the protein lengths of the Ae. Aegypti orthologs are indicated in parentheses. Tree was rooted at the midpoint for visualization purposes. Node values correspond to the percentages of 1000 ultra-fast bootstrap iterations. Branch lengths represent substitutions per site. B loqs and loqs2 exons are colored in purple and blue respectively. Black boxes represent the read mapping along loqs and loqs2 gene sequences. Lines represent intronic regions of both gene sequences and reads. *indicates species where whole genome sequencing were analyzed. Fig. S2. (Related to Fig. 2). Non-collapsed phylogenetic tree from Fig. 2B. Terminals correspond to the VEuPathDB accession numbers. Each orthology group’s dsRBDs are numbered and indicated by black bars. The tree was rooted at the midpoint for visualization purposes. Node values correspond to the percentages of 1000 ultra-fast bootstrap iterations. Branch lengths represent substitutions per site. Fig. S3. (Related to Fig. 2). Cladogram built from the Loqs-D1-Loqs2 subtree from Fig. 2B and exon–intron organization of the loqs and loqs2 genes in Ae. aegypti and Ae. albopictus. A The cladogram was built with equal branch lengths. The clade containing the Loqs2 dsRBDs is colored in grey. B The exon–intron structure of the loqs and loqs2 genes is indicated by boxes (exons) and continuous lines (introns). dsRBD regions are indicated in blue boxes. Exons are represented by boxes and introns by grey continuous lines. C Zoomed-in view of the amino-acid sequences from the exon–intron-exon boundaries within the dsRBD domains of loqs and loqs2. Fig. S4. (Related to Fig. 3). dsRBDs from L [file 12915_2024_1821_MOESM1_ESM.pdf]

Fig. S1

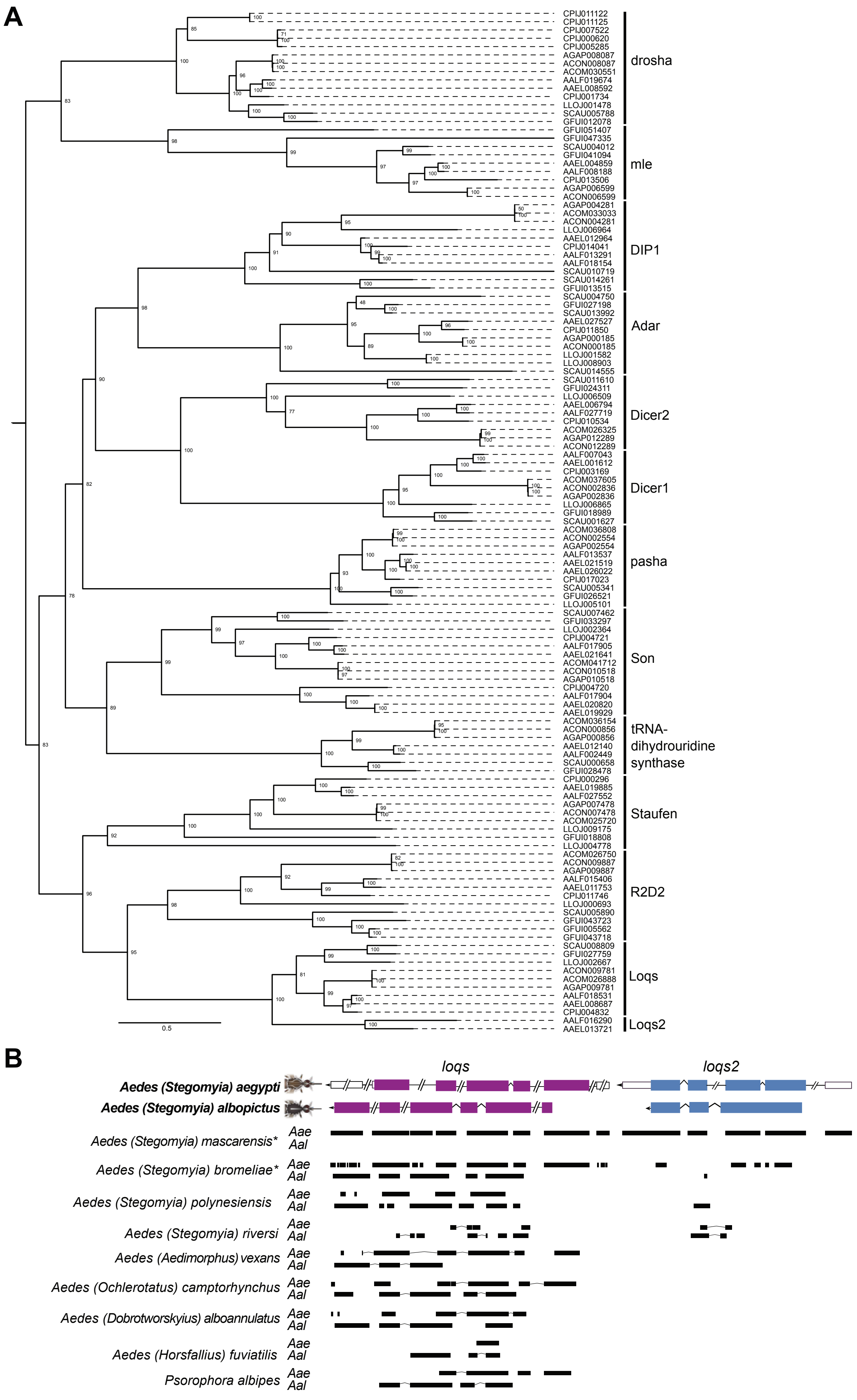

A
CPIJ011122
CPIJ011125
CPIJ007522
CPIJ000620
CPIJ005285
AGAP008087
ACON008087
ACOM030551
AALF019674
AAEL008592
CPIJ001734
LLOJ001478
SCAU005788
GFUI012078
drosha
GFUI051407
GFUI047335
SCAU004012
GFUI041094
AAEL004859
AALF008188
CPIJ013506
AGAP006599
ACON006599
mle
AGAP004281
ACOM033033
ACON004281
LLOJ006964
AAEL012964
CPIJ014041
AALF013291
AALF018154
SCAU010719
SCAU014261
GFUI013515
DIP1
SCAU004750
GFUI027198
SCAU013992
AAEL027527
CPIJ011850
AGAP000185
ACON000185
LLOJ001582
LLOJ008903
SCAU014555
Adar
SCAU011610
GFUI024311
LLOJ006509
AAEL006794
AALF027719
CPIJ010534
ACOM026325
AGAP012289
ACON012289
Dicer2
AALF007043
AAEL001612
CPIJ003169
ACOM037605
ACON002836
AGAP002836
LLOJ006865
GFUI018989
SCAU001627
Dicer1
ACOM036808
ACON002554
AGAP002554
AALF013537
AAEL021519
AAEL026022
CPIJ017023
SCAU005341
GFUI026521
LLOJ005101
pasha
SCAU007462
GFUI033297
LLOJ002364
CPIJ004721
AALF017905
AAEL021641
ACOM041712
ACON010518
AGAP010518
CPIJ004720
AALF017904
AAEL020820
AAEL019929
Son
ACOM036154
ACON000856
AGAP000856
AAEL012140
AALF002449
SCAU000658
GFUI028478
tRNA-dihydrouridine synthase
CPIJ000296
AAEL019885
AALF027552
AGAP007478
ACON007478
ACOM025720
LLOJ009175
GFUI018808
LLOJ004778
Staufen
ACOM026750
ACON009887
AGAP009887
AALF015406
AAEL011753
CPIJ011746
LLOJ000693
SCAU005890
GFUI043723
GFUI005562
GFUI043718
R2D2
SCAU008809
GFUI027759
LLOJ002667
ACON009781
ACOM026888
AGAP009781
AALF018531
AAEL008687
CPIJ004832
Loqs
AALF016290
AAEL013721
Loqs2
0.5
B
loqs
loqs2
Aedes (Stegomyia) aegypti
Aedes (Stegomyia) albopictus
Aedes (Stegomyia) mascarensis*
Aae
Aal
Aedes (Stegomyia) bromeliae*
Aae
Aal
Aedes (Stegomyia) polynesiensis
Aae
Aal
Aedes (Stegomyia) riversi
Aae
Aal
Aedes (Aedimorphus) vexans
Aae
Aal
Aedes (Ochlerotatus) camptorhynchus
Aae
Aal
Aedes (Dobrotworskyius) alboannulatus
Aae
Aal
Aedes (Horsfallius) fuviatilis
Aae
Aal
Psorophora albipes
Aae
Aal

Fig. S2

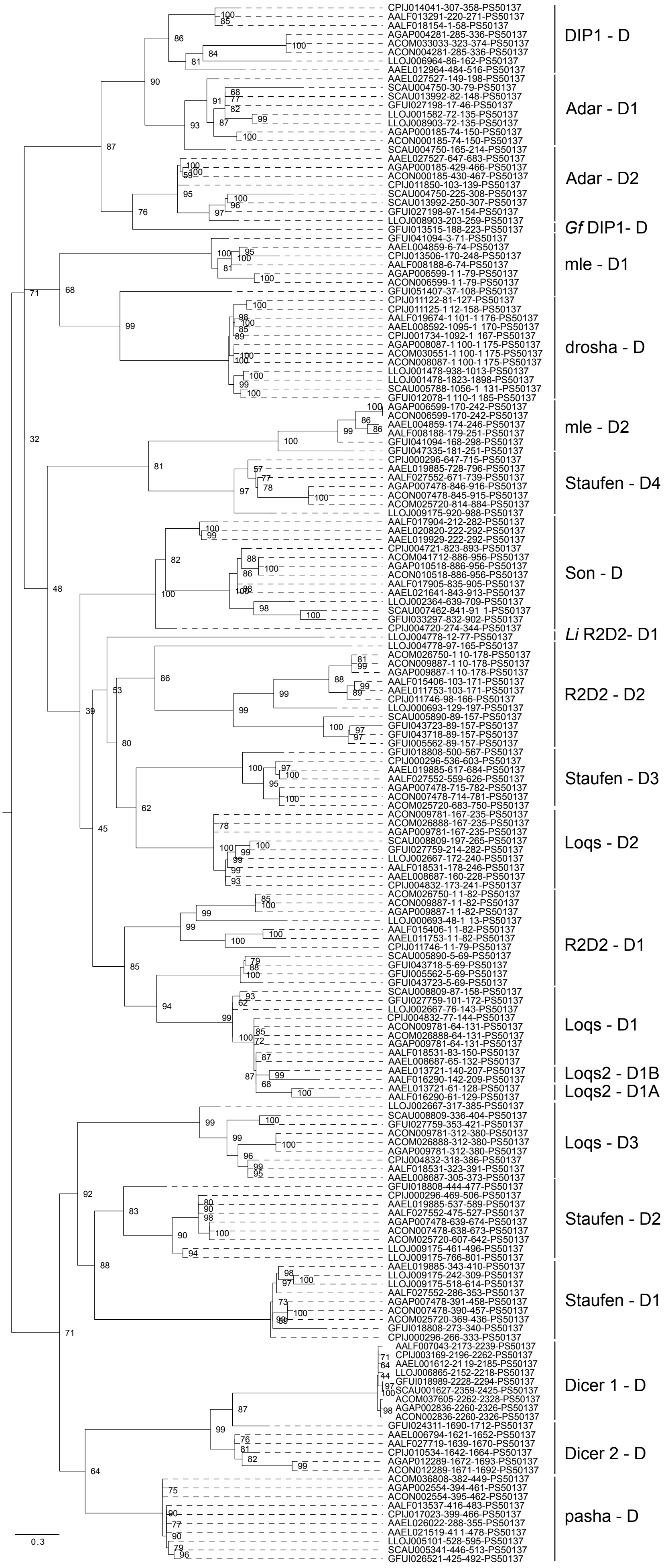

CPIJ014041-307-358-PS50137
AALF013291-220-271-PS50137
AALF018154-1-58-PS50137
AGAP004281-285-336-PS50137
ACOM033033-323-374-PS50137
ACON004281-285-336-PS50137
LLOJ006964-86-162-PS50137
AAEL012964-484-516-PS50137
DIP1 - D
AAEL027527-149-198-PS50137
SCAU004750-30-79-PS50137
SCAU013992-82-148-PS50137
GFUI027198-17-46-PS50137
LLOJ001582-72-135-PS50137
LLOJ008903-72-135-PS50137
AGAP000185-74-150-PS50137
ACON000185-74-150-PS50137
SCAU004750-165-214-PS50137
Adar - D1
AAEL027527-647-683-PS50137
AGAP000185-429-466-PS50137
ACON000185-430-467-PS50137
CPIJ011850-103-139-PS50137
SCAU004750-225-308-PS50137
SCAU013992-250-307-PS50137
GFUI027198-97-154-PS50137
LLOJ008903-203-259-PS50137
Adar - D2
GFUI013515-188-223-PS50137
Gf DIP1- D
GFUI041094-3-71-PS50137
AAEL004859-6-74-PS50137
CPIJ013506-170-248-PS50137
AALF008188-6-74-PS50137
AGAP006599-1 1-79-PS50137
ACON006599-1 1-79-PS50137
GFUI051407-37-108-PS50137
mle - D1
CPIJ011122-81-127-PS50137
CPIJ011125-1 12-158-PS50137
AALF019674-1 101-1 176-PS50137
AAEL008592-1095-1 170-PS50137
CPIJ001734-1092-1 167-PS50137
AGAP008087-1 100-1 175-PS50137
ACOM030551-1 100-1 175-PS50137
ACON008087-1 100-1 175-PS50137
LLOJ001478-938-1013-PS50137
LLOJ001478-1823-1898-PS50137
SCAU005788-1056-1 131-PS50137
GFUI012078-1 110-1 185-PS50137
drosha - D
AGAP006599-170-242-PS50137
ACON006599-170-242-PS50137
AAEL004859-174-246-PS50137
AALF008188-179-251-PS50137
GFUI041094-168-298-PS50137
GFUI047335-181-251-PS50137
mle - D2
CPIJ000296-647-715-PS50137
AAEL019885-728-796-PS50137
AALF027552-671-739-PS50137
AGAP007478-846-916-PS50137
ACON007478-845-915-PS50137
ACOM025720-814-884-PS50137
LLOJ009175-920-988-PS50137
Staufen - D4
AALF017904-212-282-PS50137
AAEL020820-222-292-PS50137
AAEL019929-222-292-PS50137
CPIJ004721-823-893-PS50137
ACOM041712-886-956-PS50137
AGAP010518-886-956-PS50137
ACON010518-886-956-PS50137
AALF017905-835-905-PS50137
AAEL021641-843-913-PS50137
LLOJ002364-639-709-PS50137
SCAU007462-841-91 1-PS50137
GFUI033297-832-902-PS50137
CPIJ004720-274-344-PS50137
Son - D
LLOJ004778-12-77-PS50137
Li R2D2- D1
LLOJ004778-97-165-PS50137
ACOM026750-1 10-178-PS50137
ACON009887-1 10-178-PS50137
AGAP009887-1 10-178-PS50137
AALF015406-103-171-PS50137
AAEL011753-103-171-PS50137
CPIJ011746-98-166-PS50137
LLOJ000693-129-197-PS50137
SCAU005890-89-157-PS50137
GFUI043723-89-157-PS50137
GFUI043718-89-157-PS50137
GFUI005562-89-157-PS50137
R2D2 - D2
GFUI018808-500-567-PS50137
CPIJ000296-536-603-PS50137
AAEL019885-617-684-PS50137
AALF027552-559-626-PS50137
AGAP007478-715-782-PS50137
ACON007478-714-781-PS50137
ACOM025720-683-750-PS50137
Staufen - D3
ACON009781-167-235-PS50137
ACOM026888-167-235-PS50137
AGAP009781-167-235-PS50137
SCAU008809-197-265-PS50137
GFUI027759-214-282-PS50137
LLOJ002667-172-240-PS50137
AALF018531-178-246-PS50137
AAEL008687-160-228-PS50137
CPIJ004832-173-241-PS50137
Loqs - D2
ACOM026750-1 1-82-PS50137
ACON009887-1 1-82-PS50137
AGAP009887-1 1-82-PS50137
LLOJ000693-48-1 13-PS50137
AALF015406-1 1-82-PS50137
AAEL011753-1 1-82-PS50137
CPIJ011746-1 1-79-PS50137
SCAU005890-5-69-PS50137
GFUI043718-5-69-PS50137
GFUI005562-5-69-PS50137
GFUI043723-5-69-PS50137
R2D2 - D1
SCAU008809-87-158-PS50137
GFUI027759-101-172-PS50137
LLOJ002667-76-143-PS50137
CPIJ004832-77-144-PS50137
ACON009781-64-131-PS50137
ACOM026888-64-131-PS50137
AGAP009781-64-131-PS50137
AALF018531-83-150-PS50137
AAEL008687-65-132-PS50137
Loqs - D1
AAEL013721-140-207-PS50137
AALF016290-142-209-PS50137
Loqs2 - D1B
AAEL013721-61-128-PS50137
AALF016290-61-129-PS50137
Loqs2 - D1A
LLOJ002667-317-385-PS50137
SCAU008809-336-404-PS50137
GFUI027759-353-421-PS50137
ACON009781-312-380-PS50137
ACOM026888-312-380-PS50137
AGAP009781-312-380-PS50137
CPIJ004832-318-386-PS50137
AALF018531-323-391-PS50137
AAEL008687-305-373-PS50137
Loqs - D3
GFUI018808-444-477-PS50137
CPIJ000296-469-506-PS50137
AAEL019885-537-589-PS50137
AALF027552-475-527-PS50137
AGAP007478-639-674-PS50137
ACON007478-638-673-PS50137
ACOM025720-607-642-PS50137
LLOJ009175-461-496-PS50137
LLOJ009175-766-801-PS50137
Staufen - D2
AAEL019885-343-410-PS50137
LLOJ009175-242-309-PS50137
LLOJ009175-518-614-PS50137
AALF027552-286-353-PS50137
AGAP007478-391-458-PS50137
ACON007478-390-457-PS50137
ACOM025720-369-436-PS50137
GFUI018808-273-340-PS50137
CPIJ000296-266-333-PS50137
Staufen - D1
AALF007043-2173-2239-PS50137
CPIJ003169-2196-2262-PS50137
AAEL001612-2119-2185-PS50137
LLOJ006865-2152-2218-PS50137
GFUI018989-2228-2294-PS50137
SCAU001627-2359-2425-PS50137
ACOM037605-2262-2328-PS50137
AGAP002836-2260-2326-PS50137
ACON002836-2260-2326-PS50137
Dicer 1 - D
GFUI024311-1690-1712-PS50137
AAEL006794-1621-1652-PS50137
AALF027719-1639-1670-PS50137
CPIJ010534-1642-1664-PS50137
AGAP012289-1672-1693-PS50137
ACON012289-1671-1692-PS50137
Dicer 2 - D
ACOM036808-382-449-PS50137
AGAP002554-394-461-PS50137
ACON002554-395-462-PS50137
AALF013537-416-483-PS50137
CPIJ017023-399-466-PS50137
AAEL026022-288-355-PS50137
AAEL021519-41 1-478-PS50137
LLOJ005101-528-595-PS50137
SCAU005341-446-513-PS50137
GFUI026521-425-492-PS50137
pasha - D
0.3

Fig. S3

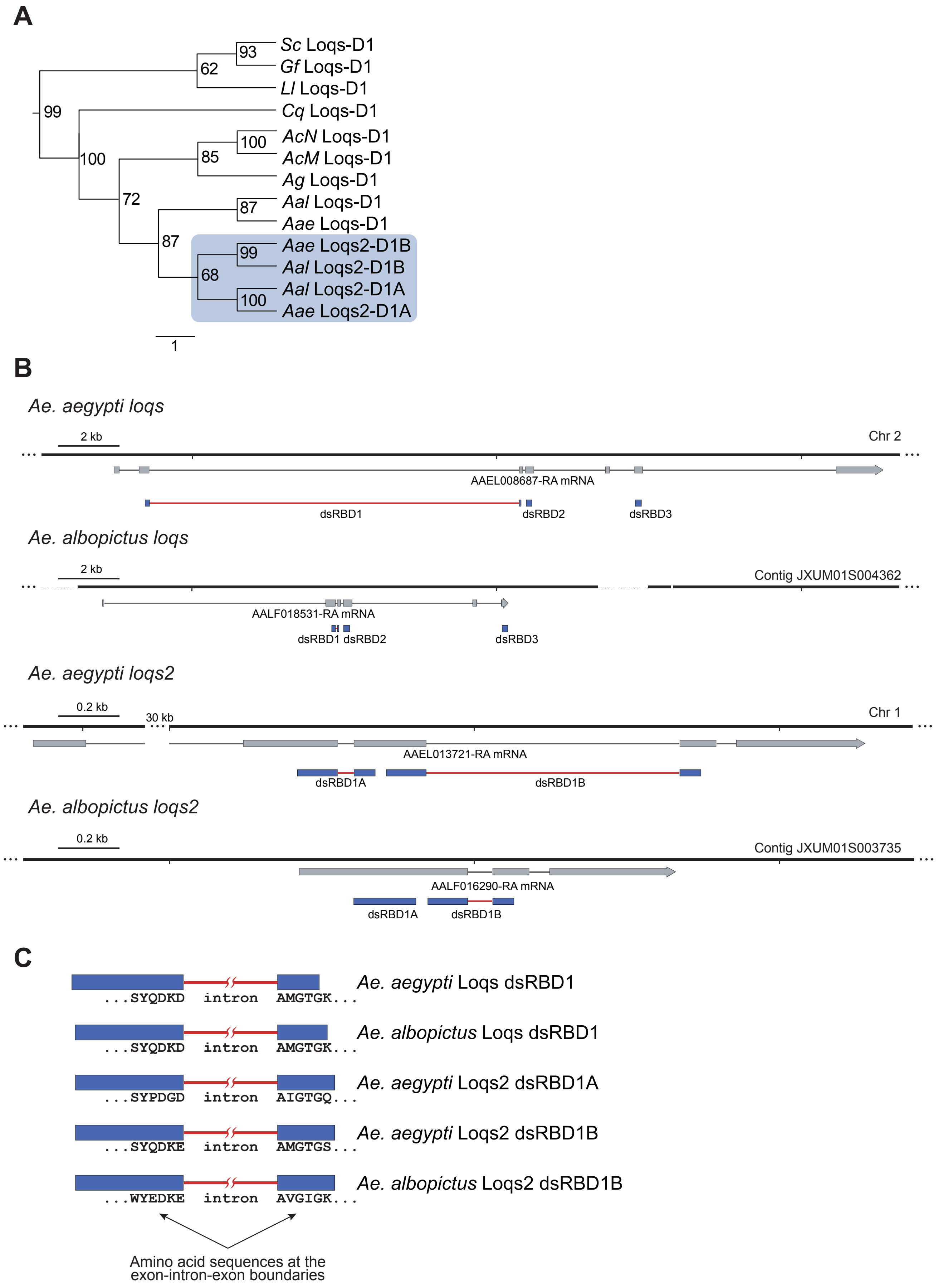
A
Sc Loqs-D1
Gf Loqs-D1
Ll Loqs-D1
Cq Loqs-D1
AcN Loqs-D1
AcM Loqs-D1
Ag Loqs-D1
Aal Loqs-D1
Aae Loqs-D1
Aae Loqs2-D1B
Aal Loqs2-D1B
Aal Loqs2-D1A
Aae Loqs2-D1A
93
62
99
100
100
85
72
87
87
99
68
100
1
B
Ae. aegypti loqs
2 kb
Chr 2
AAEL008687-RA mRNA
dsRBD1
dsRBD2
dsRBD3
Ae. albopictus loqs
2 kb
Contig JXUM01S004362
AALF018531-RA mRNA
dsRBD1 dsRBD2
dsRBD3
Ae. aegypti loqs2
0.2 kb
30 kb
Chr 1
AAEL013721-RA mRNA
dsRBD1A
dsRBD1B
Ae. albopictus loqs2
0.2 kb
Contig JXUM01S003735
AALF016290-RA mRNA
dsRBD1A
dsRBD1B
C
...SYQDKD intron AMGTGK...
Ae. aegypti Loqs dsRBD1
...SYQDKD intron AMGTGK...
Ae. albopictus Loqs dsRBD1
...SYPDGD intron AIGTGQ...
Ae. aegypti Loqs2 dsRBD1A
...SYQDKE intron AMGTGS...
Ae. aegypti Loqs2 dsRBD1B
...WYEDKE intron AVGIGK...
Ae. albopictus Loqs2 dsRBD1B
Amino acid sequences at the
exon-intron-exon boundaries

Fig. S4

A

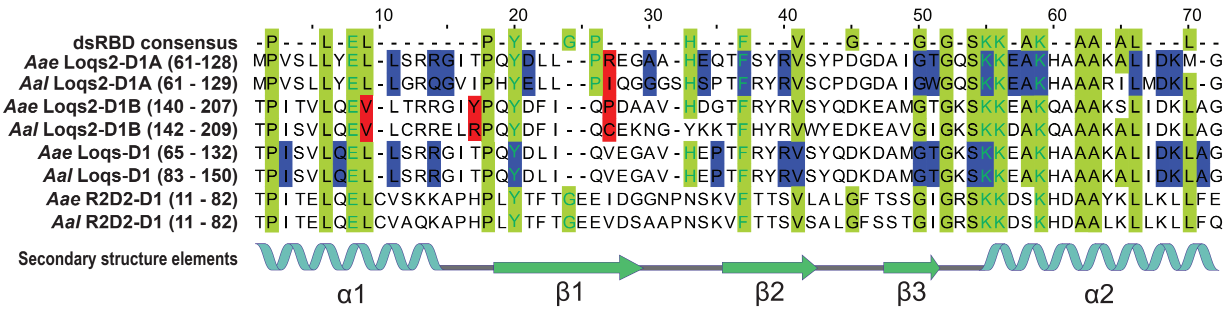
10 20 30 40 50 60 70
dsRBD consensus -P---L-EL---------P-Y---G-P------H---F---V---G----G-G-SKK-AK--AA-AL---L--
Aae Loqs2-D1A (61-128) MPVSLLYEL-LSRRGITPQYDLL--PREGAA-HEQTFSYRVSYPDGDAIGTGQSKKEAKHAAAKALIDKM-G
Aal Loqs2-D1A (61 - 129) MPVSLLYEL-LGRQGVIPHYELL--PIQGGGSHSPTFRYRVSCPDGDAIGWGQSKKEAKHAAARILMDKL-G
Aae Loqs2-D1B (140 - 207) TPITVLQEV-LTRRGIYPQYDFI--QPDAAV-HDGTFRYRVSYQDKEAMGTGKSKKEAKQAAAKSLIDKLAG
Aal Loqs2-D1B (142 - 209) TPISVLQEV-LCRRELRPQYDFI--QCEKNG-YKKTFHYRVWYEDKEAVGIGKSKKDAKQAAAKALIDKLAG
Aae Loqs-D1 (65 - 132) TPISVLQEL-LSRRGITPQYDLI--QVEGAV-HEPTFRYRVSYQDKDAMGTGKSKKEAKHAAAKALIDKLAG
Aal Loqs-D1 (83 - 150) TPISVLQEL-LSRRGITPQYDLI--QVEGAV-HEPTFRYRVSYQDKDAMGTGKSKKEAKHAAAKALIDKLAG
Aae R2D2-D1 (11 - 82) TPITELQELCVSKKAPHPLYTFTGEEIDGGNPNSKVFTTSVLALGFTSSGIGRSKKDSKHDAAYKLLKLLFE
Aal R2D2-D1 (11 - 82) TPITELQELCVAQKAPHPLYTFTGEEVDSAAPNSKVFTTSVSALGFSSTGIGRSKKDSKHDAALKLLKLLFQ
Secondary structure elements
α1
β1
β2
β3
α2

Fig. S5

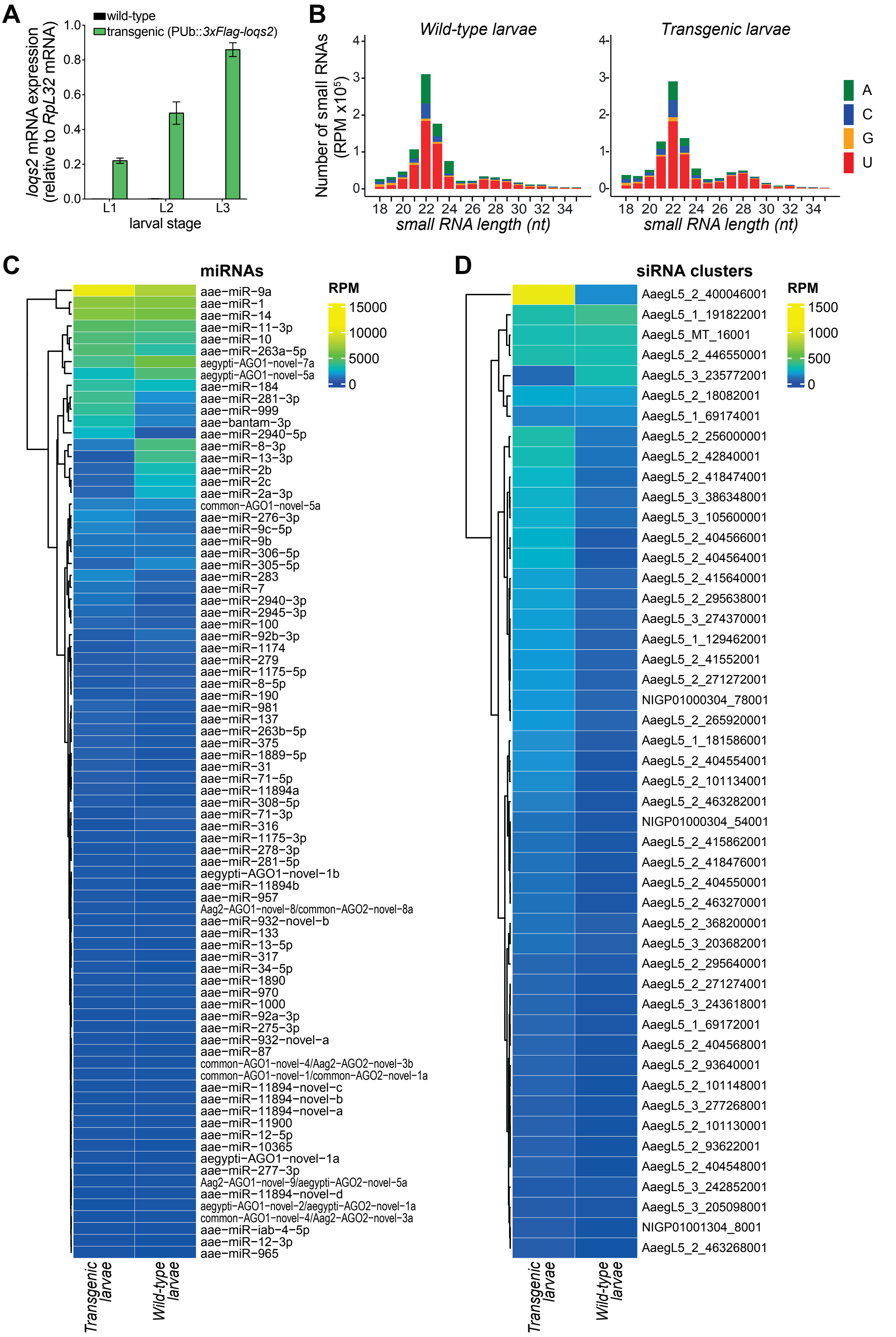

A
wild-type
transgenic (PUb::3xFlag-loqs2)
loqs2 mRNA expression (relative to RpL32 mRNA)
1.0
0.8
0.6
0.4
0.2
0.0
L1
L2
L3
larval stage
B
Wild-type larvae
Transgenic larvae
Number of small RNAs (RPM x10^5)
small RNA length (nt)
A
C
G
U
C
miRNAs
RPM
15000
10000
5000
0
aae-miR-9a
aae-miR-1
aae-miR-14
aae-miR-11-3p
aae-miR-10
aae-miR-263a-5p
aegypti-AGO1-novel-7a
aegypti-AGO1-novel-5a
aae-miR-184
aae-miR-281-3p
aae-miR-999
aae-bantam-3p
aae-miR-2940-5p
aae-miR-8-3p
aae-miR-13-3p
aae-miR-2b
aae-miR-2c
aae-miR-2a-3p
common-AGO1-novel-5a
aae-miR-276-3p
aae-miR-9c-5p
aae-miR-9b
aae-miR-306-5p
aae-miR-305-5p
aae-miR-283
aae-miR-7
aae-miR-2940-3p
aae-miR-2945-3p
aae-miR-100
aae-miR-92b-3p
aae-miR-1174
aae-miR-279
aae-miR-1175-5p
aae-miR-8-5p
aae-miR-190
aae-miR-981
aae-miR-137
aae-miR-263b-5p
aae-miR-375
aae-miR-1889-5p
aae-miR-31
aae-miR-71-5p
aae-miR-11894a
aae-miR-308-5p
aae-miR-71-3p
aae-miR-316
aae-miR-1175-3p
aae-miR-278-3p
aae-miR-281-5p
aegypti-AGO1-novel-1b
aae-miR-11894b
aae-miR-957
Aag2-AGO1-novel-8/common-AGO2-novel-8a
aae-miR-932-novel-b
aae-miR-133
aae-miR-13-5p
aae-miR-317
aae-miR-34-5p
aae-miR-1890
aae-miR-970
aae-miR-1000
aae-miR-92a-3p
aae-miR-275-3p
aae-miR-932-novel-a
aae-miR-87
common-AGO1-novel-4/Aag2-AGO2-novel-3b
common-AGO1-novel-1/common-AGO2-novel-1a
aae-miR-11894-novel-c
aae-miR-11894-novel-b
aae-miR-11894-novel-a
aae-miR-11900
aae-miR-12-5p
aae-miR-10365
aegypti-AGO1-novel-1a
aae-miR-277-3p
Aag2-AGO1-novel-9/aegypti-AGO2-novel-5a
aae-miR-11894-novel-d
aegypti-AGO1-novel-2/aegypti-AGO2-novel-1a
common-AGO1-novel-4/Aag2-AGO2-novel-3a
aae-miR-iab-4-5p
aae-miR-12-3p
aae-miR-965
Transgenic larvae
Wild-type larvae
D
siRNA clusters
RPM
1500
1000
500
0
AaegL5_2_400046001
AaegL5_1_191822001
AaegL5_MT_16001
AaegL5_2_446550001
AaegL5_3_235772001
AaegL5_2_18082001
AaegL5_1_69174001
AaegL5_2_256000001
AaegL5_2_42840001
AaegL5_2_418474001
AaegL5_3_386348001
AaegL5_3_105600001
AaegL5_2_404566001
AaegL5_2_404564001
AaegL5_2_415640001
AaegL5_2_295638001
AaegL5_3_274370001
AaegL5_1_129462001
AaegL5_2_41552001
AaegL5_2_271272001
NIGP01000304_78001
AaegL5_2_265920001
AaegL5_1_181586001
AaegL5_2_404554001
AaegL5_2_101134001
AaegL5_2_463282001
NIGP01000304_54001
AaegL5_2_415862001
AaegL5_2_418476001
AaegL5_2_404550001
AaegL5_2_463270001
AaegL5_2_368200001
AaegL5_3_203682001
AaegL5_2_295640001
AaegL5_2_271274001
AaegL5_3_243618001
AaegL5_1_69172001
AaegL5_2_404568001
AaegL5_2_93640001
AaegL5_2_101148001
AaegL5_3_277268001
AaegL5_2_101130001
AaegL5_2_93622001
AaegL5_2_404548001
AaegL5_3_242852001
AaegL5_3_205098001
NIGP01001304_8001
AaegL5_2_463268001
Transgenic larvae
Wild-type larvae

Fig. S6

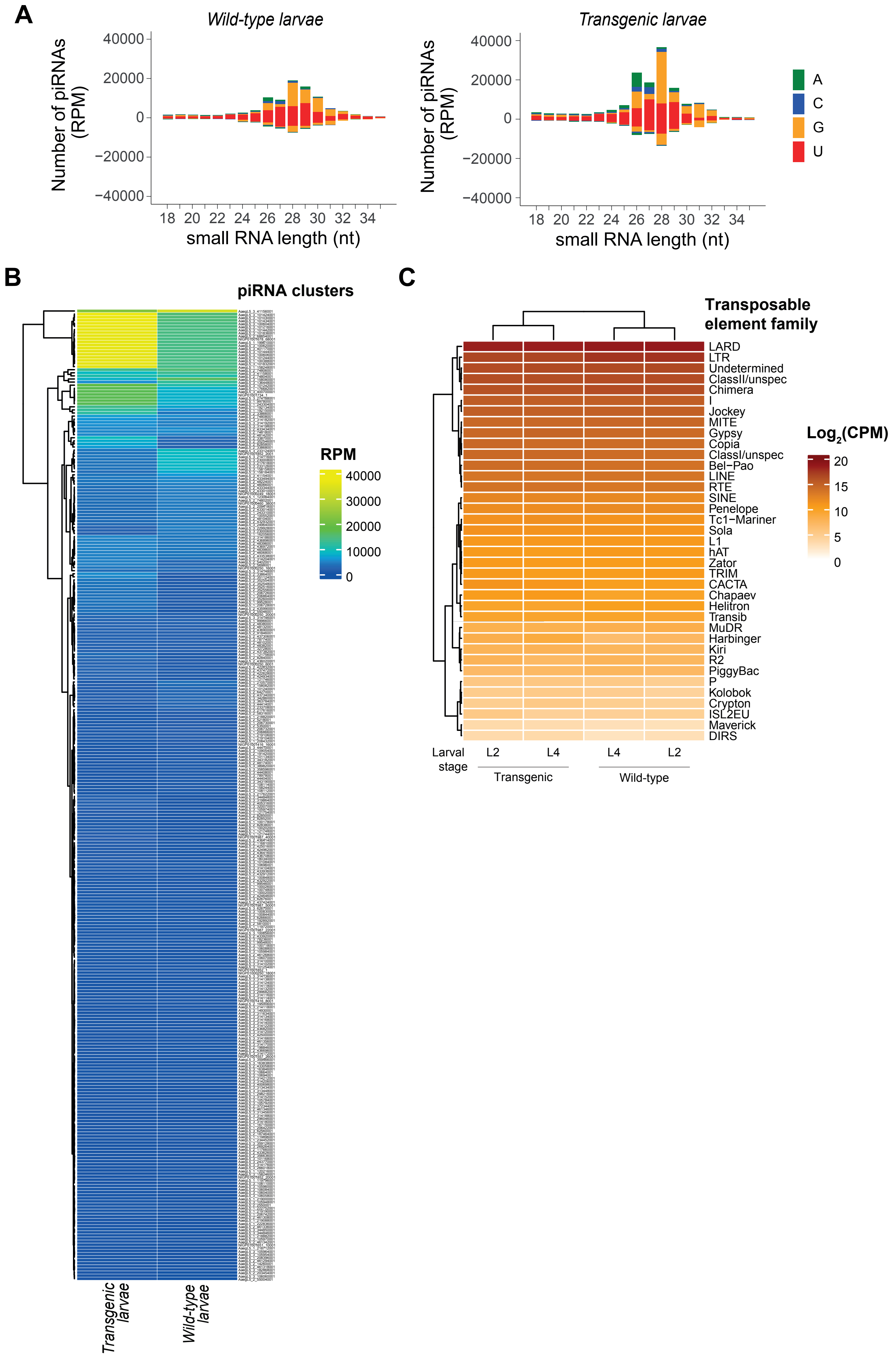
A
Wild-type larvae
Transgenic larvae
Number of piRNAs (RPM)
40000
20000
0
−20000
−40000
18 20 22 24 26 28 30 32 34
small RNA length (nt)
A
C
G
U
B
piRNA clusters
RPM
40000
30000
20000
10000
0
Transgenic larvae
Wild-type larvae
C
Transposable element family
LARD
LTR
Undetermined
ClassII/unspec
Chimera
I
Jockey
MITE
Gypsy
Copia
ClassI/unspec
Bel−Pao
LINE
RTE
SINE
Penelope
Tc1−Mariner
Sola
L1
hAT
Zator
TRIM
CACTA
Chapaev
Helitron
Transib
MuDR
Harbinger
Kiri
R2
PiggyBac
P
Kolobok
Crypton
ISL2EU
Maverick
DIRS
Log2(CPM)
20
15
10
5
0
Larval stage
L2
L4
L4
L2
Transgenic
Wild-type

Fig. S7

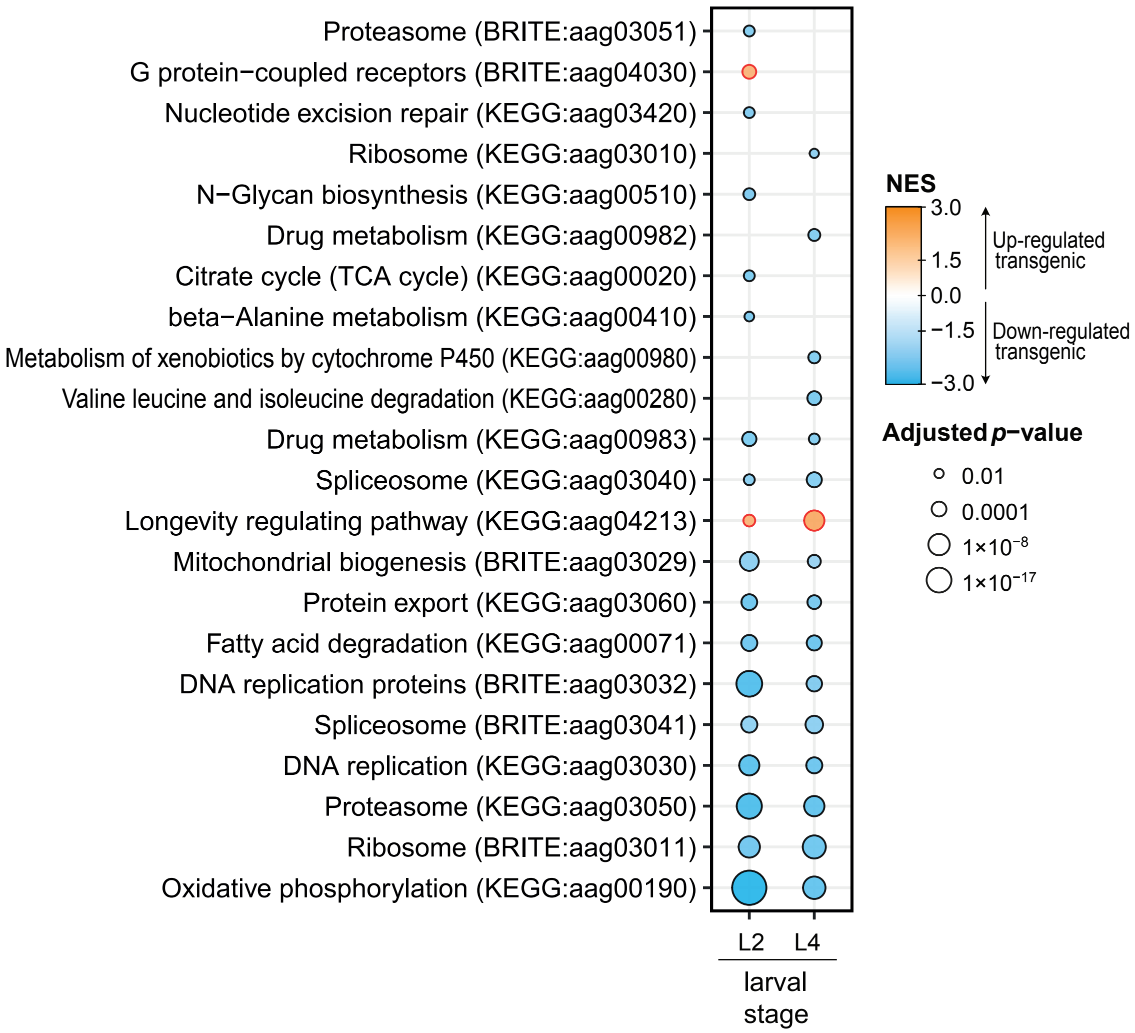
Proteasome (BRITE:aag03051)
G protein−coupled receptors (BRITE:aag04030)
Nucleotide excision repair (KEGG:aag03420)
Ribosome (KEGG:aag03010)
N−Glycan biosynthesis (KEGG:aag00510)
Drug metabolism (KEGG:aag00982)
Citrate cycle (TCA cycle) (KEGG:aag00020)
beta−Alanine metabolism (KEGG:aag00410)
Metabolism of xenobiotics by cytochrome P450 (KEGG:aag00980)
Valine leucine and isoleucine degradation (KEGG:aag00280)
Drug metabolism (KEGG:aag00983)
Spliceosome (KEGG:aag03040)
Longevity regulating pathway (KEGG:aag04213)
Mitochondrial biogenesis (BRITE:aag03029)
Protein export (KEGG:aag03060)
Fatty acid degradation (KEGG:aag00071)
DNA replication proteins (BRITE:aag03032)
Spliceosome (BRITE:aag03041)
DNA replication (KEGG:aag03030)
Proteasome (KEGG:aag03050)
Ribosome (BRITE:aag03011)
Oxidative phosphorylation (KEGG:aag00190)
L2
L4
larval stage
NES
3.0
1.5
0.0
−1.5
−3.0
Up-regulated transgenic
Down-regulated transgenic
Adjusted p−value
0.01
0.0001
1×10⁻⁸
1×10⁻¹⁷
